# Bone Response to Fluoride Exposure Is Influenced by Genetics

**DOI:** 10.1371/journal.pone.0114343

**Published:** 2014-12-11

**Authors:** Cláudia A. N. Kobayashi, Aline L. Leite, Camila Peres-Buzalaf, Juliane G. Carvalho, Gary M. Whitford, Eric T. Everett, Walter L. Siqueira, Marília A. R. Buzalaf

**Affiliations:** 1 Department of Biological Sciences, Bauru School of Dentistry, University of São Paulo, Bauru, SP, Brazil; 2 Centro de Ciências da Saúde, Universidade do Sagrado Coração, Bauru, SP, Brazil; 3 Department of Oral Biology, College of Dental Medicine, Georgia Regents University, Augusta, Georgia, United States; 4 Department of Pediatric Dentistry, The Carolina Center for Genome Sciences, School of Dentistry, University of North Carolina, Chapel Hill, North Carolina, United States; 5 Schulich School of Medicine and Dentistry, Western University, London, ON, Canada; Oklahoma State University, United States of America

## Abstract

Genetic factors influence the effects of fluoride (F) on amelogenesis and bone homeostasis but the underlying molecular mechanisms remain undefined. A label-free proteomics approach was employed to identify and evaluate changes in bone protein expression in two mouse strains having different susceptibilities to develop dental fluorosis and to alter bone quality. *In vivo* bone formation and histomorphometry after F intake were also evaluated and related to the proteome. Resistant 129P3/J and susceptible A/J mice were assigned to three groups given low-F food and water containing 0, 10 or 50 ppmF for 8 weeks. Plasma was evaluated for alkaline phosphatase activity. Femurs, tibiae and lumbar vertebrae were evaluated using micro-CT analysis and mineral apposition rate (MAR) was measured in cortical bone. For quantitative proteomic analysis, bone proteins were extracted and analyzed using liquid chromatography-electrospray ionization-tandem mass spectrometry (LC-ESI-MS/MS), followed by label-free semi-quantitative differential expression analysis. Alterations in several bone proteins were found among the F treatment groups within each mouse strain and between the strains for each F treatment group (ratio ≥1.5 or ≤0.5; *p*<0.05). Although F treatment had no significant effects on BMD or bone histomorphometry in either strain, MAR was higher in the 50 ppmF 129P3/J mice than in the 50 ppmF A/J mice treated with 50 ppmF showing that F increased bone formation in a strain-specific manner. Also, F exposure was associated with dose-specific and strain-specific alterations in expression of proteins involved in osteogenesis and osteoclastogenesis. In conclusion, our findings confirm a genetic influence in bone response to F exposure and point to several proteins that may act as targets for the differential F responses in this tissue.

## Introduction

Fluoride (F) is readily absorbed from the stomach and small intestine, followed by its deposition in calcified tissues and urinary excretion [1]. The anabolic effect of F on bone mass has been studied for many years, but there is still controversy about the quality of the bone formed [2], [3]. Evaluation of the efficacy of F therapy is complicated, since it can have a biphasic dose-dependent response on bone cells, i.e., stimulating bone formation at a low dose and being toxic at a high dose [4], [5]. The effectiveness of F therapy for osteoporosis could depend on earlier administration of low-dose regimens in which toxic levels are avoided and mineralization is not affected [6].

Although the dose of F is highly relevant, it is not the only factor influencing the effects of F on bone. Approximately one third of patients receiving F therapy for osteoporosis are non-responders [7]. Furthermore, about 40% of the population living in areas with naturally high F levels in water were not affected by skeletal fluorosis [8] and 5% of children in high F areas had no dental fluorosis (DF) [9]. The sensitivity of trabecular bone to both anabolic and catabolic stimuli is influenced by genetics and this could explain, in part, why treatments with F for low bone mass are not universally effective [10].

The influence of genetic background in F-induced responses is also supported by studies comparing different mouse strains. Differences in DF susceptibility/resistance among 12 inbred strains of mice have been observed [11]. Compared to the other strains tested, the A/J strain was highly susceptible, rapidly developing severe DF, while the 129P3/J strain was more resistant, exhibiting only slight degrees of DF [11], [12]. The A/J and 129P3/J mice also have a differential bone response to F. Increasing F doses (0, 25, 50 and 100 ppm) for these mouse strains had different effects on the mechanical properties of both cortical and trabecular bone. Mechanical testing showed remarkable reduction in bone quality in the A/J strain after receiving F, whereas no significant effect in the 129P3/J strain was observed [13]. This was not related to a reduction in F uptake by bone, since previous work from our laboratory has shown that the femur F concentrations were higher in the 129P3/J strain than in the A/J strain [12]. In additional study, beyond the physical and chemical effects on bone mineral, treatment with 100 ppmF in drinking water significantly increased osteoid volume and osteoid area for both strains [14]. The increase in osteoid formation supports the observation that F induces osteoblastic activity and delays mineralization of newly formed bone.

The present study was designed to investigate the effects of F on bone and the molecular mechanisms that could account for the effects. We used label-free quantitative proteomic analysis complemented by static and dynamic histomorphometry. We chose the A/J and 129P3/J strains of mice as the models since, as discussed above, they have been shown to have distinctly different responses to fluoride exposure. We concluded that F exposure at high levels enhanced bone formation in 129P3/J mice, but not in A/J mice, confirming a genetic influence in the response of bone to F. Several proteins could account for the strain-specific skeletal phenotype after F exposure.

## Materials and Methods

### Mice and Fluoride Treatment

The research protocol was approved by the Institutional Animal Care and Use Committee of the University of São Paulo, Bauru Dental School. Weanling (21 days old) male 129P3/J and A/J mice were obtained from the Jackson Laboratory (Bar Harbor, ME, USA) and randomly distributed into three groups given low-F diet (0.95 ppm F, AIN76A, PMI Nutrition, Richmond, IN, USA) and water containing 10 or 50 ppmF added as NaF for 8 weeks. They led to plasma F levels corresponding to those found in humans drinking water containing 1 (optimally fluoridated areas) and 10 mg/L F (areas of endemic fluorosis), respectively [15]. Control groups received water without F. Each group consisted of 32 mice, 16 from each strain. Plasma was collected for alkaline phosphatase (ALP) activity. Femurs, tibiae, and lumbar vertebrae were harvested, and cleaned of soft tissues. Some bones were fixed in 95% v/v ethanol and stored in 70% v/v ethanol at 4°C. Others were snap frozen in liquid N_2_ and stored at −80°C until used.

### Micro-CT analysis

Femurs, tibiae and 4th lumbar vertebrae were subjected to micro-CT analysis (n = 8/group). Bones were scanned using the Skyscan 1074HR microCT (Skyscan, Aartselaar, Belgium) at the resolution of 20.7 µm/pixel. Standardized scanning and image reconstruction settings fully automated were used. Hydroxyapatite phantoms (250 mg/cc and 750 mg/cc) (CIRS, Inc., Norfolk, VA) were used to determine bone mineral densities within regions and volumes of interest. Morphometric parameters were calculated by CT-analyser (v1.9.1.0, Skyscan, Kontich, Belgium) either in 3D based on a volume model, or in 2D from cross-sectional images. Trabecular regions of the proximal tibiae and L4 vertebrae were used to determine bone mineral density (BMD), bone volume fraction (BV/TV), specific bone surface (BS/BV), bone surface density (BS/TV), trabecular thickness (Tb.Th), trabecular separation (Tb.Sp), trabecular number (Tb.N), and trabecular bone pattern factor (Pb.Pf). One mm cortical regions within the mid-diaphysis of the femurs were used to determine mean total cross-sectional bone area (B.Ar), mean total cross-sectional bone perimeter (B.Pm), mean total cross-sectional tissue area (T.Ar), mean total cross-sectional tissue perimeter (T.Pm), mean polar moment of inertia (MMI), and cortical BMD.

### Mineral Apposition Rate (MAR)

Mice (n = 8/group) were injected intraperitoneally with calcein (10 mg/Kg in 2% sodium bicarbonate, 0.1 mL/mouse) at 7 days prior to euthanasia and Alizarin Complexone (30 mg/Kg in 2% sodium bicarbonate, 0.1 mL/mouse) 2 days prior to euthanasia. Bones were dried to touch and embedded in epoxy, Pelco Eponate 12 (25.7 mL resin +9.3 mL DDSA +16.5 mL NMA +1.6 mL BDMA) (Ted Pella, Inc., Redding, CA, USA). After 24 h cure at 60°C, samples were sawed using a Buehler Isomet Low Speed saw (Buehler Inc., Lake Bluff, IL, USA) equipped with a diamond wafer saw blade and 200–300 micron thick sections were cemented to glass slides. Mounted sections were ground using 600-C silicon carbide wet paper (Mager Scientific Inc., Dexter, MI, USA) to a final thickness of 100 microns. Calcein and alizarin complexone fluorophores were visualized at 100X and 400X using epifluorescence microscopy, Nikon Eclipse 50i equipped with a XF52 dual band filter set (excitation at 490 and 550, dichroic 490–550 and emitter at 520 and 580) (Omega Optical, Brattleboro, VT, USA). Images were captured using a Nikon DXM1200 camera and ACT-1 software (Nikon Instruments Inc., Melville, NY, USA).

### Protein Extraction/Sample preparation

Protein extraction was performed according to the method described by Domenicucci et al. [16] with a slight modification. Frozen femurs (n = 8/group) were ground with a mortar and pestle. All of the following steps were performed at 4°C with constant stirring. The bone chips were washed in PBS with protease inhibitors (three times for a total of 24 h) to extract bone marrow proteins. After centrifugation, the supernatant was collected as PBS Extract. The pellet was treated four times with G-buffer (4 M guanidine HCl, 50 mM Tris/HCl, pH 7.4 containing protease inhibitors). The supernatant was collected as G1 Extract after centrifugation. The residue was then washed three times in PBS containing protease inhibitors, followed by four times steps of extraction with E- buffer every 24 hours each (0.5 M EDTA, 50 mM-Tris/HCl, pH 7.4 containing protease inhibitors) to release mineral-associated proteins. After centrifugation the supernatant was collected as EDTA Extract. Finally, the pellet was washed three times in PBS with protease inhibitors and was then extracted again with G-buffer four times over 96 h. The supernatant was collected as G2 Extract after centrifugation. To remove the excess of guanidine HCl, G1G2 extracts were cleaned up using PD MIniTrap G-10 (GE Healthcare, Munich, Germany). Excess of EDTA and other interfering substances was removed from samples by centrifugal filtration using the Amicon Ultra-4 Centrifugal Filter Unit with Ultracel-3 membrane (Millipore, Billerica, MA). These extracts were pooled and protein concentration was determined by the Pierce BCA protein assay.

### LC MS/MS

For each group, 300 µg of protein were denatured and reduced in a buffer containing 100 µL of 4 M urea, 10 mM DTT and 50 mM NH_4_HCO_3_, pH 7.8 for 1 h at room temperature (RT). Then, the samples were diluted to 1 M urea with 50 mM NH_4_HCO_3_, pH 7.8. Finally, trypsin (Trypsin Gold, Promega, Madison, WI) was added (1∶20 w/w), and the solution incubated at 37°C for 18 h. The extracted peptides were concentrated and desalted with Perfect Pure C18 Tips (Eppendorf, Hamburg, Germany) and analyzed by LC-ESI-MS/MS. The Nanoflow HPLC/MS/MS system consists of a Proxeon EasyNano-LC (ThermoFisher, USA) coupled to LTQ-VELOS-Ion Trap (ThermoFisher, USA) MS. The nano-LC system includes a 5 mm L.×0.3 mm I.D. reverse phase trap column packed with 5 µm C18 Zorbax 300SB beads (Agilent, USA), and nanoflow analytical column (150 mm L.×0.075 mm I.D. with 0.015 mm tip opening) packed with the same reversed phase C18 beads as in the Trap column. Peptides were eluted from the column using a linear gradient profile from 6% Solvent B to 65% solvent B in 30 min, followed by 3 min wash with 95% solvent B (Solvent A: 2% acetonitrile with 0.1% formic acid; Solvent B: 98% acetonitrile with 0.1% formic acid). The flow rate was set to 300 nL/min with a maximum pressure of 280 bar. Electrospray voltage and the temperature of the ion transfer capillary were 1.8 kV and 250°C respectively. All samples were analyzed in triplicate, resulting in 18 LC-MS/MS runs. The acquired MS/MS spectra was searched against the *Mus musculus* database from NCBI (Version Dec/2010) using SEQUEST algorithm in Proteome Discoverer 1.3 software (Thermo Scientific, San Jose, CA, USA) for protein identification. The minimum number of peptides to identify proteins was set at two. Search results were filtered for a False Discovery rate of 1% employing a decoy search strategy utilizing a reverse database. An additional inclusion criterion for positive identification of proteins was the same protein passing the filter score in at least two different MS analyses from the same time-point group in a total of three MS analyses per group. The label-free semi-quantitative differential expression analysis was performed with SIEVE software 1.3 (Thermo Fisher Scientific, San Jose, CA). Changes in relative protein abundance between the groups (A/B) were regarded as significant when the ratio was ≤0.5 for increase in abundance in B compared to A (B>A) or ≥1.5 for decrease in abundance in B compared to A (B<A), and a *p*-value <0.05. Identified bone proteins were classified according to their biological processes and cellular localization using Web-based Babelomics (http://babelomics.bioinfo.cipf.es/index.html), MGI Mouse Genome Informatics (http://www.informatics.jax.org/), and Uniprot protein data base (http://www.informatics.jax.org/).

### Plasma ALP activity

The ALP activity was measured in plasma by enzymatic assay using p-nitrophenyl phosphate (pNPP) as substrate. For the ALP activity assay, 100 µL of a solution containing 25 mM glycine buffer (pH 9.4), 2 mM MgCl_2_ and 1 mM p-NPP were added to 96-well plates. After incubation for 30 min in a water bath, 5 µL of each sample (in duplicate) were added. The plate was kept at 37°C for 60 min. Then the reaction was stopped with 1 M NaOH. The final product (p-nitrophenol) was quantified at 405 nm (ε = 18,000 M^−1^ cm^−1^) and the results were expressed as specific activity (nmol p-nitrophenol min^−1^ mg^−1^ of protein).

### Western blotting

Four independent experiments were performed with individual sample of each group, totalizing 4 different samples per group. Mouse femur samples were extracted as described above. For each group, 30 µg of protein were separated on 10% SDS-PAGE gels and transferred onto a PVDF membrane. After blocking with 1% low-fat milk for 1 h at RT, the membranes were incubated at 4°C overnight with rabbit polyclonal anti-mouse collagen type I (Millipore, AB765P) and anti-β-tubulin (Santa Cruz Biotechnology, sc-9104) in 3% BSA and 0.1% TBS-Tween (TBS-T). The membranes were then washed 3 times for 10 min with 0.1% TBS-T, and incubated for 1 hour at RT with horseradish peroxidase-conjugated secondary antibody (GE Healthcare, Buckinghamshire, UK). After washing, proteins of interest were detected using the enhanced chemiluminescence technique (ECL) Plus Western Blotting Detection Reagent (GE Healthcare, NJ, USA) according to the manufacturer's recommendations, followed by exposure to x-ray film (Amersham Hyperfilm ECL, GE Healthcare). Quantification was performed by densitometric analysis using Image J software (available at http://rsbweb.nih.gov/ij/).

### Statistical analysis

The software GraphPad InStat version 4.0 for Windows (GraphPad Software Inc., La Jolla, CA, USA) and Statistica version 7.0 for windows (Stat Soft Inc., Tulsa, USA) were used for all statistical analyzes. Since the assumptions of equality of variance (Bartlett test) and normal distribution of errors (Kolmogorov-Smirnov test) were satisfied, two-way ANOVA and Bonferroni's post hoc tests were carried out for statistical comparisons. In all cases, statistical significance was defined as a *p* value of <0.05. The data are expressed as mean ± SD.

## Results

### Impact of F in the bone architecture

**Figure 1:**
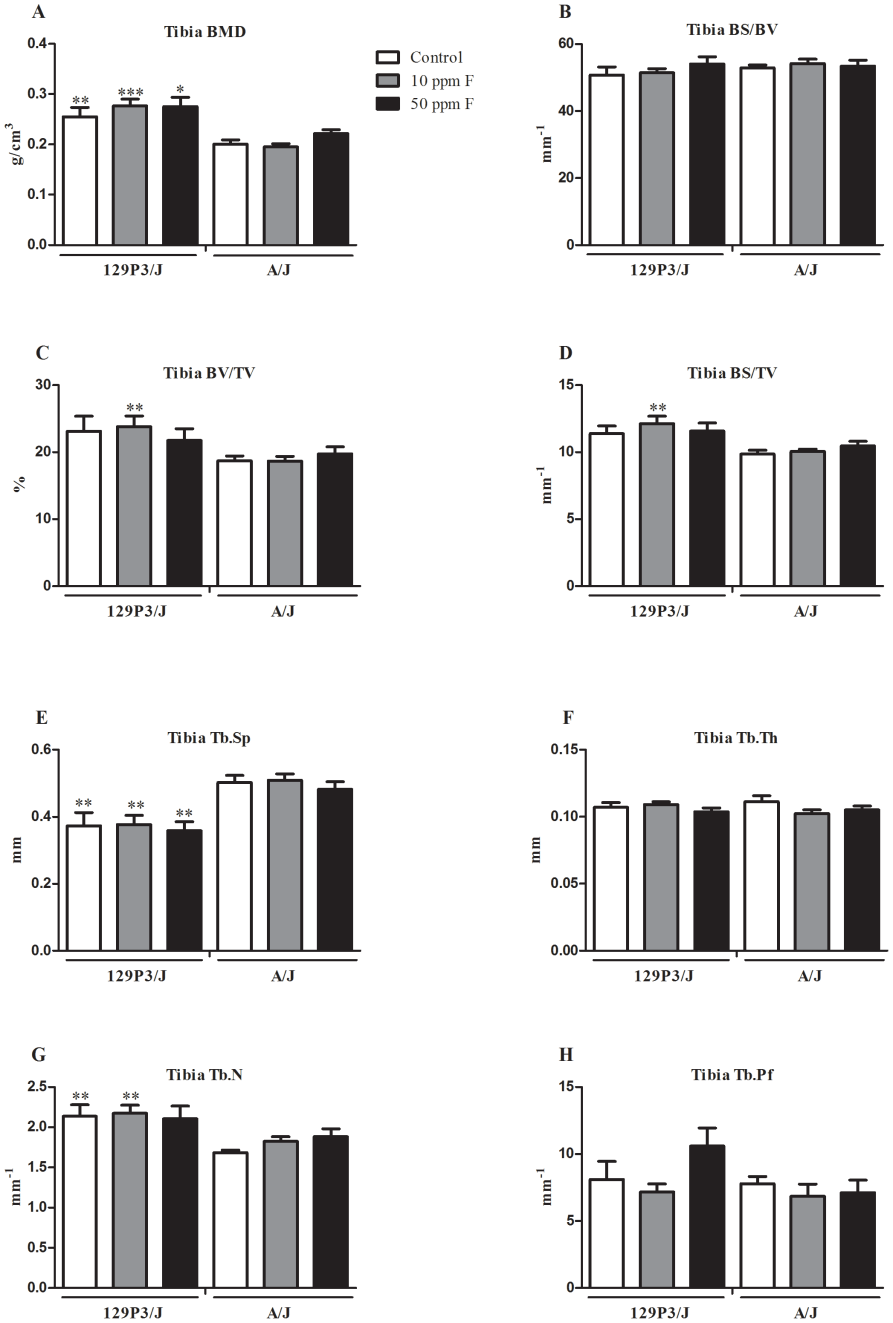
BMD and histomorphometric parameters in trabecular region of tibiae from A/J and 129P3/J mice treated with 0, 10 or 50 ppm F in drinking water for 8 weeks. Values are mean ± SD, n = 8/group. A =  Bone mineral density (BMD); B =  Specific bone surface (BS/BV); C =  Bone volume fraction (BV/TV); D =  Bone surface density (BS/TV); E =  Trabecular separation (Tb.Sp); F =  Trabecular thickness (Tb.Th); G =  Trabecular number (Tb.N); H =  Trabecular bone pattern factor (Tb.Pf). *p<0.05, **p<0.01 and *** *p*<0.001 represents significant differences between strains, for each group. No statisticallly significant differences were found for each strain after receiving F.

**Figure 2:**
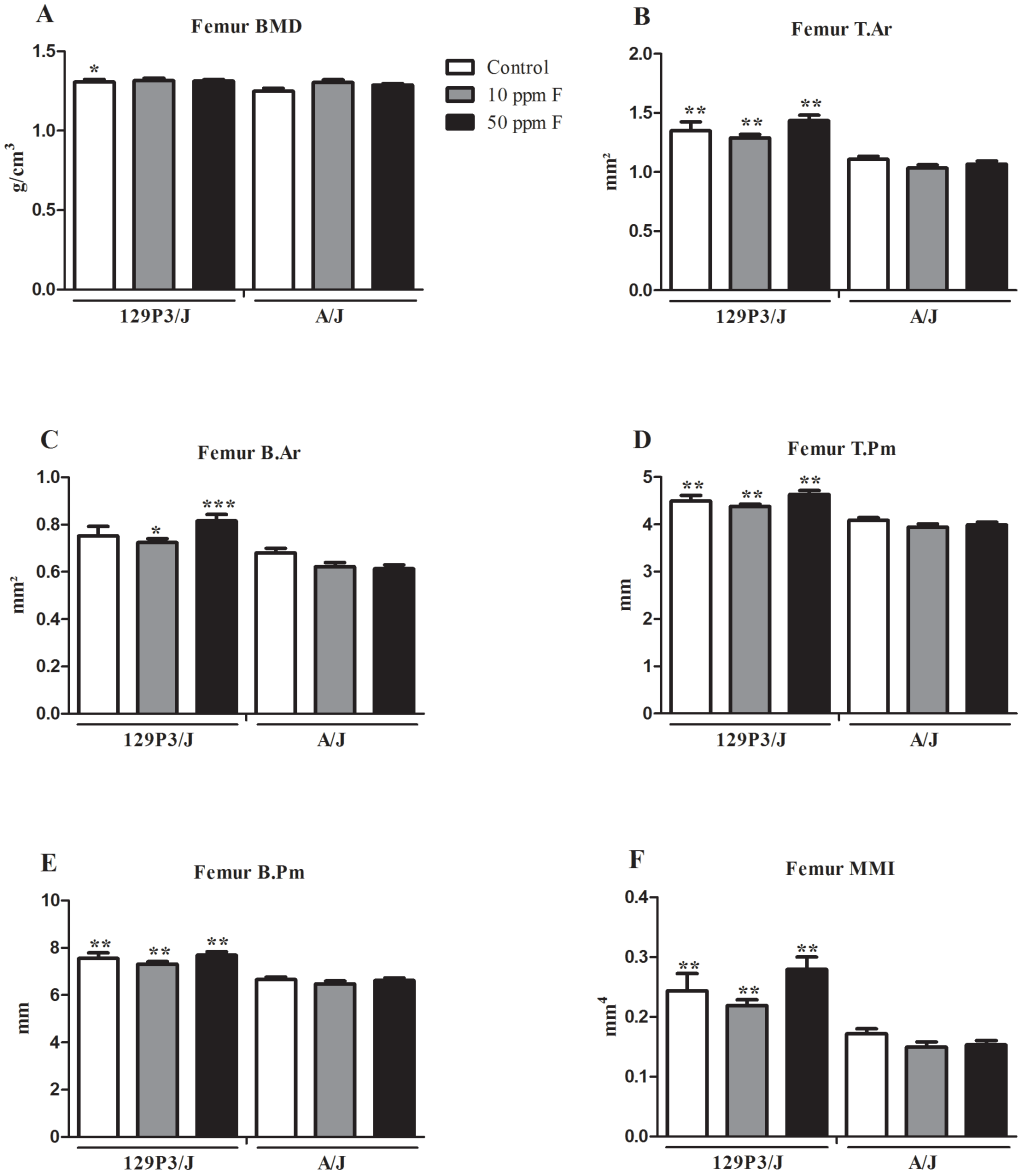
BMD and histomorphometric parameters in cortical region of femur from A/J and 129P3/J mice treated with 0, 10 or 50 ppm F. Values are mean ± SD, n = 8/group. A =  Bone mineral density (BMD); B =  Mean total cross-sectional tissue area (T.Ar); C =  Mean total cross-sectional bone area (B.Ar); D =  Mean total cross-sectional tissue perimeter T.Pm); E =  Mean total cross-sectional bone perimeter (B.Pm); F =  Mean polar moment of inertia (MMI). *p<0.05, **p<0.01 and *** *p*<0.001 represents significant differences between strains, for each group. No statisticallly significant differences were found for each strain after receiving F.

To assess trabecular and cortical bone morphology after treatment with F we have conducted tibia and femur micro-CT, respectively. The lumbar vertebra was also employed for the trabecular study since it has been shown to be a target for the F effect (S1 Figure) [14]. There were no significant differences in BMD among the F-treated groups for any bone in either strain (Figs. 1*A*, 2*A*, S1*A* Figure). The treatment with F did not alter any trabecular or cortical bone parameters in either strain (Figs. 1 and 2). The tibia BMD was higher in 129P3/J than A/J, independent of F treatment and dosage (*p*<0.01, *p*<0.001 and *p*<0.05 for control, 10 and 50 ppmF groups, respectively) while femur BMD was higher only in control group (*p*<0.05) (Figs. 1*A* and 2*A*). Tibia and vertebral trabecular bone quantity and structure were also significantly different between the two strains. BS/TV and BV/TV in 129P3/J were higher than those of the A/J strain for all F treatment groups, but the difference was only between 10 ppmF-treated groups for tibia (*p*<0.01) (Fig. 1*B,C*) and among all groups for the vertebrae (*p*<0.01) (S1*B*,*C* Figure). Tb.N was higher in 129P3/J than A/J in control and 10 ppm F-treated groups for tibia (*p*<0.01) (Fig. 1*G*) and in all groups for vertebrae (*p*<0.01) (S1*E* Figure). No differences were found for Tb.Th in tibia whereas it was enhanced in vertebrae from 10 and 50 ppmF-treated 129P3/J (*p*<0.005 and *p*<0.002, respectively) (S1*F* Figure). On the other hand, Tb.Sp and Tb.Pf were decreased in tibia and vertebrae from 129P3/J compared to A/J, independent of treatment (*p*<0.01) (Fig. 1*E*, S1*G, H* Figure). In addition, all bone cortical parameters were higher in femur from 129P3/J than A/J mice (Fig. 2). Statistical differences in B.Pm, T.Pm, MMI and T.Ar were seen among all groups (*p*<0.01) (Fig. 2*B,D,E,F*) and in B.Ar when comparing F-treated groups (Fig. 2*C*) (*p*<0.05, *p*<0.001, respectively).

### F enhances bone formation in 129P3/J but not in A/J

**Figure 3:**
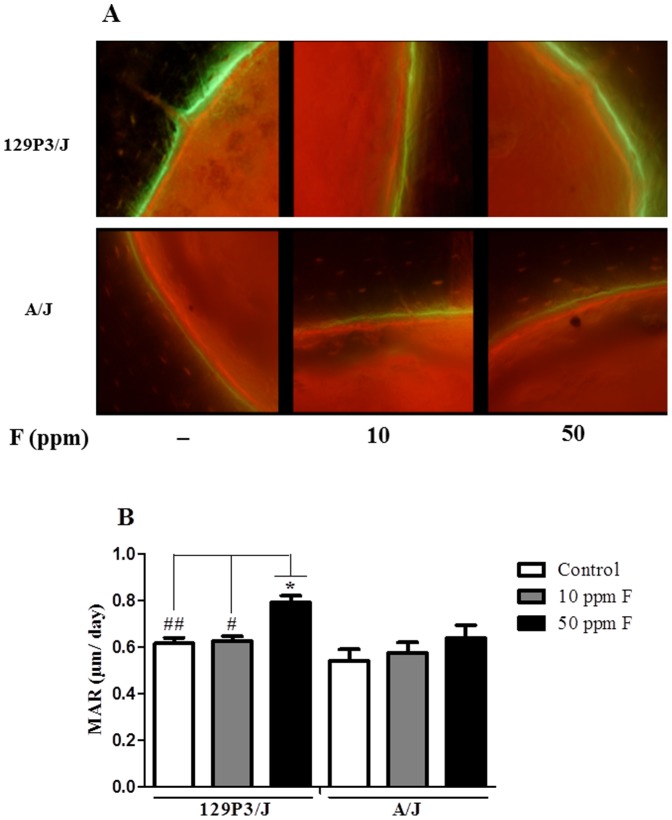
Mineral apposition rate (MAR) of femur from A/J and 129P3/J mice after F exposure. (A) Upper squares: Representative images showing calcein (green) and alizarin complexone (red) labeled mid-diaphyseal cross-sections of 129P3/J mice. Lower squares: Representative images showing calcein (green) and alizarin complexone (red) labeled mid-diaphyseal cross-sections of A/J mice. Concentrations of F are indicated. (B) Values are mean ± SD of the measurement distances between the labels, n = 8/group. ^*^ Represents significant differences between strains for each group (*p*<0.05); ^#^ *p*<0.01 and ^##^ *p*<0.001 represent significant differences of control and 10 ppmF-treated 129P3/J versus 50 ppmF-treated 129P3/J mice, respectively.

Slight dose-dependent increases in the rate of new bone deposition were observed for both strains but they were significant only in the 50 ppmF-treated 129P3/J group when compared to the control and 10 ppmF groups (*p*<0.01 and *p*<0.05, respectively) (Fig. 3*A,B*). Comparing both strains, the MAR data were significantly higher in 50 ppmF-treated 129P3/J than the respective A/J (*p*<0.05) (Fig. 3*A,B*).

### Plasma ALP activity

**Figure 4:**
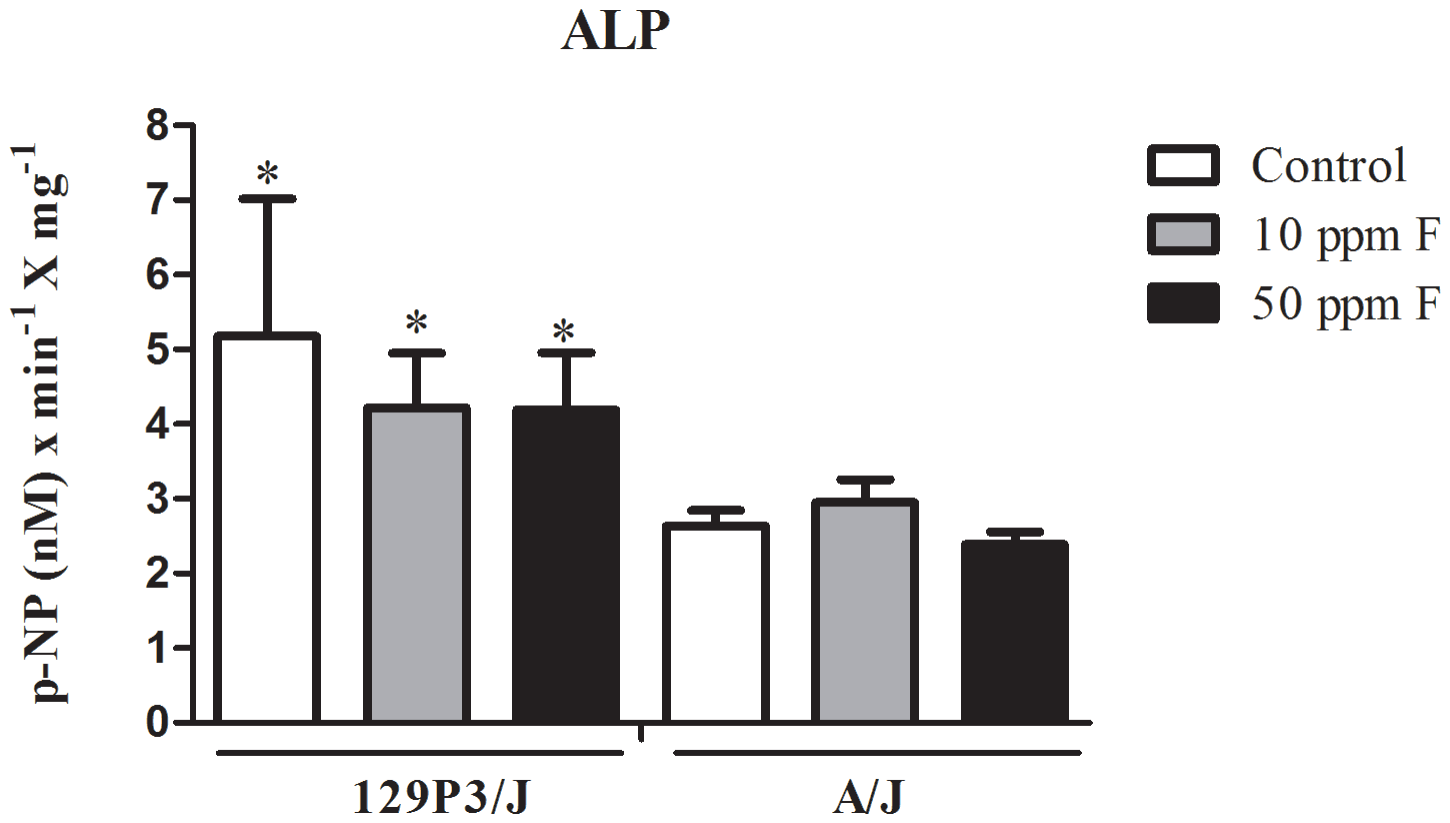
Quantification of ALP activity in plasma from A/J and 129P3/J mice treated with 0, 10 or 50 ppm F in the drinking water for 8 weeks. Results are shown as mean ± SD of enzymatic activity (nmol of p-NP per min per mg of total protein). ^*^Represents significant differences between strains for each group (*p*<0.05). No statistical differences were found for each strain after receiving F.

We used plasma biochemical assay to investigate alterations in bone modeling and response to F in both strains. Total ALP activity, as an indirect marker for bone ALP activity, was unaffected. No statistical differences were observed among the F treatments for either strain. However, plasma ALP activity in the 129P3/J strain was significantly higher compared to the A/J strain for all groups (F = 6.59, *p* = 0.014) (Fig. 4).

### Quantitative proteomic analysis of bone

A total of nine differential analyses for relative protein abundance was performed with SIEVE software 1.3 (Thermo Fisher Scientific, San Jose, CA), three comparisons among the F treatments for 129P3/J strain, three among the F treatments for A/J strain and three between the strains for each F treatment. For the 129P3/J strain, comparisons between control and experimental groups showed an increase in level of 29 and 126 proteins in 10 ppmF and 50 ppmF groups, respectively, while only 1 protein decreased in level in 50 ppmF group (Tables 1, S1 and S2 Tables). Comparisons of the 129P3/J experimental groups showed an increase and decrease in the levels of 2 and 3 proteins, respectively, in the 50 ppmF group compared to 10 ppmF group (Table 1 and S3 Table). For the A/J strain, comparisons of the control and experimental groups showed increases in levels of 35 and 1 proteins and decreases in levels of 1 and 16 proteins in 10 ppmF and 50 ppmF groups, respectively (Table 1, S4 and S5 Tables). Comparisons between F-treated A/J groups showed a decrease in the levels of 15 proteins in 50 ppm F with respect to 10 ppm F group (Table 1 and S6 Table). Comparing the strains, 163 proteins were more abundant in 129P3/J control group compared to A/J control group (Table 1 and S7 Table). The 129P3/J 10 ppmF group showed 8 and 6 proteins more and less abundant, respectively, compared to 10 ppmF-treated A/J group (Table 1 and S8 Table). Finally, 4 proteins were more and only 1 protein was less abundant in the 129P3/J 50 ppmF group compared to its respective group in A/J (Table 1 and S9 Table).

**Table 1:** Total number of identified bone proteins with differences in abundance in each comparison, by label-free semi-quantitative differential expression analysis.

Groups of comparison A×B	Total number of proteins identified	Number of proteins with differential abundance	Number of proteins (ratio A/B≥1,5)*	Number of proteins (ratio A/B≤0,5)*
129P3/J control ×129P3/J 10 ppm F	1316	29	0	29
129P3/J control ×129P3/J 50 ppm F	1391	127	1	126
129P3/J 10 ppm F ×129P3/J 50 ppm F	1196	5	2	3
A/J control ×A/J 10 ppm F	1455	36	1	35
A/J control ×A/J 50 ppm F	1369	17	16	1
A/J 10 ppm F ×A/J 50 ppm F	1437	15	15	0
129P3/J control ×A/J control	1449	163	163	0
A/J 10 ppm F ×129P3/J 10 ppm F	1321	14	6	8
A/J 50 ppm F ×129P3/J 50 ppm F	644	5	4	1

*Ratio of the relative protein abundance between each comparison. Significant differences in protein abundance were considered when ratio ≤0.5 or ≥1.5. Ratios of ≤0.5 or ≥1.5 mean increase or decrease in group B in relation to group A, respectively.

Proteins with differences in abundance in each comparison were classified according to their biological process and cellular location (S2–S5 Figures). Several proteins such as exportin-2, NADPH oxidase 4 (Nox-4), collagen alpha-2(I) chain, protocadherin beta 15, secreted frizzled-related protein-4 (FRP-4), bone sialoprotein 2 and bone morphogenetic protein 1 (BMP-1) presented their abundance increased in bone of control 129P3/J compared to control A/J mice (Table 2 and S7 Table). The treatment of 129P3/J mice with 50 ppmF promoted an enhancement of Nox-1, chromodomain helicase DNA binding protein 4 (CDH-4) and -7 (CDH-7), protocadherin beta 9, catenin alpha-2 and phosphatidylinositol 3-4-5 trisphosphate 5-phosphatase 2 (SHIP-2), aflatoxin B1 aldehyde reductase member 2 (AFB1-AR) and carbonyl reductase NADPH 2 (CBR) compared to untreated control mice (Table 3 and S2 Table). Moreover, the treatment of A/J with 50 ppmF increased the abundance of SHIP-1 while it diminished the abundance of exportin-2 (Table 4 and S5 Table).

**Table 2:** Summary of identified proteins with differences in abundance in the comparison between control 129P3/J and control A/J mice.

Acession Number^a^	Protein^b^	Ratio^c^	N° of peptides^d^
Q9ERK4	Exportin-2	1.6	2
Q9JHI8	NADPH oxidase 4	2.7	2
Q01149	Collagen alpha-2(I) chain	1.5	3
Q91Y04	Protocadherin beta 15	1.5	2
P11087	Collagen alpha-1(I) chain	1.5	6
Q9Z1N6	Secreted frizzled-related sequence protein 4	2.3	2
Q61711	Bone sialoprotein 2	1.5	2
P98063	Bone morphogenetic protein 1	1.5	3

a Protein accession numbers from UniProtKB.

b Protein name.

c Ratio of the relative protein abundance between (A) control 129P3/J and (B) control A/J mice. Significant differences in protein abundance were considered when ratio ≤0.5. Ratio of ≤0.5 means increase in group B in relation to group A.

d Number of peptides identified.

**Table 3:** Summary of identified proteins with differences in abundance in the comparison between control 129P3/J and 50 ppmF-treated 129P3/J mice.

Acession Number^a^	Protein^b^	Ratio^c^	N° of peptides^d^
Q9JHI8	NADPH oxidase 4	0.2	2
A2AJK6	Chromodomain-helicase-DNA-binding protein 7	0.5	2
Q6PDQ2	Chromodomain-helicase-DNA-binding protein 4	0.4	4
Q91XZ1	Protocadherin beta 9	0.5	2
Q61301	Catenin alpha-2	0.2	2
Q6P549	Phosphatidylinositol 3,4,5-trisphosphate 5-phosphatase 2	0.4	3
Q8CG76	Aflatoxin B1 aldehyde reductase member 2	0.5	2
P08074	Carbonyl reductase [NADPH] 2	0.5	2

a Protein accession numbers from UniProtKB.

b Protein name.

c Ratio of the relative protein abundance between (A) control 129P3/J and (B) control A/J mice. Significant differences in protein abundance were considered when ratio ≤0.5. Ratio of ≤0.5 means increase in group B in relation to group A.

d Number of peptides identified.

**Table 4:** Summary of identified proteins with differences in abundance in the comparison between control A/J and 50 ppmF-treated A/J mice.

Acession Number^a^	Protein^b^	Ratio^c^	N° of peptides^d^
Q7TQC8	Eukaryotic translation initiation factor 2 alpha kinase 3	1.5	2
Q9ERK4	Exportin-2	1.5	2
Q9ES52	Phosphatidylinositol 3,4,5-trisphosphate 5-phosphatase 1	0.4	2

a Protein accession numbers from UniProtKB.

b Protein name.

c Ratio of the relative protein abundance between (A) control 129P3/J and (B) control A/J mice. Significant differences in protein abundance were considered when ratio ≤0.5. Ratio of ≤0.5 means increase in group B in relation to group A.

d Number of peptides identified.

### Confirmation of collagen type I expression by Western blotting

**Figure 5:**
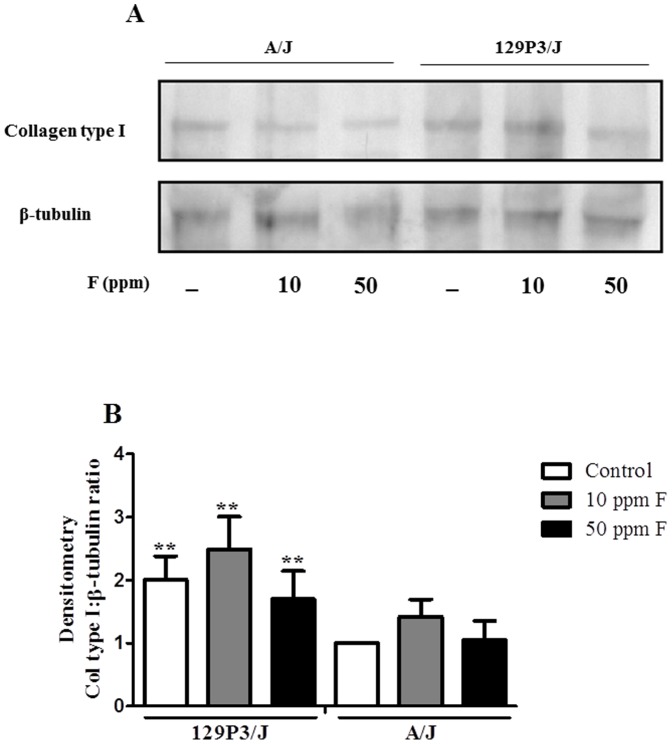
Western blot analysis of collagen type I. (A) Representative immunoblot showing collagen type I levels (upper) in A/J and 129P3/J mice after treatment with 0, 10 or 50 ppmF in drinking water for 8 weeks. The level of β-tubulin was used as control of sample loading (lower). (B) Values are mean ± SD of arbitrary values of four independent experiments quantified by densitometry analysis using the Image J Software (NIH Image). Arbitrary values from control A/J were standardized as 1. ^**^Represents significant differences between strains for each treatment (*p*<0.01). No statistical differences were found for each strain after receiving F.

Analysis of collagen type I expression in mouse femur corroborated the quantitative proteomics data, confirming that there were no statistically significant differences among the F treatments for either strain (F = 1.45, *p* = 0.262) but there was a significant increase in the level of collagen type I in 129P3/J compared to A/J strain (F = 9.54, *p* = 0.006) for all F treatment groups (Fig. 5*A,B*). However, it is worth mentioning that quantitative proteomic analysis identified a significant difference between the strains only for control group.

## Discussion

We have shown that A/J and 129P3/J strains present inherent differences in cortical and trabecular bone and that they are not affected by F treatment. Strain-specific differences are related to the BMD, bone surface to-tissue volume ratio, bone volume-to-tissue volume ratio, trabecular number, thickness, and separation, revealing that A/J mice presented fewer, thinner, less connected, and more widely spaced trabeculae than those in 129P3/J. Cortical microstructure parameters in the femur are also diminished in A/J mice. In addition, plasma ALP activity, an enzyme involved in the mineralization process, is enhanced in 129P3/J mice. Although exposure to F either failed to change [17] or increased BMD [1], [9], our data indicate that lumbar vertebrae of A/J mice subjected to mechanical tests are less resistant than those of 129P3/J mice, regardless of F treatment [13].

Fluorescent bone markers were also used to investigate the dynamic process of bone formation. In both strains, F promoted a slightly enhanced rate of bone formation, whereas the treatment of 129P3/J with 50 ppmF group presented significantly higher MAR, compared to other groups. In combination with phenytoin, F at 50 ppm in the drinking water promoted an increase of osteocalcin level, bone ALP specific activity, MAR, bone formation rate and mineralizing surface in adult rats, showing its effectiveness in stimulating bone formation *in vivo* [17]. Although both histomorphometry and MAR data seem to be contradictory, they do not rule out the possibility that F promotes increased bone formation in 129P3/J. A possible explanation is based on the fact that the new bone may not be totally mineralized, thus compromising the mineral content and mineral density at that time. Further evaluation of static histomorphometric parameters over prolonged periods would clarify this matter.

Analysis of LC-ESI MS/MS data revealed differences in protein abundance among F treatments and between the strains of mice. We have focused on identified proteins that may be associated with bone cellular metabolism. Collagen type I (collagen alpha 1 and 2 (I) chain precursors), the major protein of bone, was more abundant in control 129P3/J mice compared to control A/J mice. These data were further confirmed by western blotting analysis. Since mechanical properties of bone tissue depend both on the mineral and matrix (primarily type I collagen fibrils) constituents, as well as their geometrical arrangement, the greater abundance of collagen type I in the 129P3/J strain could explain the higher mechanical strength [13] observed in the femurs of 129P3/J strain compared to the A/J strain. Also, the increased levels of bone sialoprotein 2, a non-collagen bone protein that mediates mineral deposition, and bone morphogenetic protein (BMP)-1 in 129P3/J may contribute to the greater strain-specific bone formation. These data are consistent with the enhancement of osteoid formation in 129P3/J mice compared to A/J mice, regardless of F treatment [14].

Exportin-2 (CSE1L) is a protein transport that mediates importin-alpha re-export from the nucleus to the cytosol after import substrates have been released into the nucleoplasm [18]. Its abundance in control 129P3/J mice is higher compared to control A/J mice. Similarly, exportin-1 is involved in the transport of Cbfa1, a transcriptional factor essential for osteogenesis [19]. In the present study, the greater abundance of exportin-2 in mature bone of 129P3/J mice may be evidence that this similar transport factor is involved in the increased strain dependent-osteoblast differentiation. Although sFRPs are known as negative modulators of Wnt signaling, some isoforms such as sFRP-4, up-regulated in bone of control 129P3/J mice compared to A/J mice, were shown to increase β-catenin/TCR reporter activity followed by ALP activity [20]. Based on these data, this protein could also be involved in the enhanced bone formation in these mice.

CHD7 was more abundant in 129P3/J mice and, along with CHD4, it was up-modulated by 50 ppmF. Although no role has been reported for CHD-4 and CHD-7 in bone, another chromatin remodeling protein, namely CReMM/CHD9, is expressed by osteoprogenitors cells in mature bone and regulates gene promoters of osteocalcin [21]. These proteins may play an unexplored role in mediating transcriptional responses involved in osteoblast function. Moreover, since cadherin-catenin interactions are crucial for intercellular adhesion [22], enhanced levels of both in 50 ppmF-treated 129P3/J mice also suggest the positive impact of F in promoting strain-specific osteoblast differentiation.

Proteins associated with endoplasmatic reticulum (ER) stress, such as eukaryotic translation initiation factor 2 alpha kinase 3 precursor (eIF2αk3) or PKR-like endoplasmic reticulum kinase (PERK), were also affected by F. ER stress during osteoblast differentiation activates the PERK-eIF2α-ATF4 signaling pathway involved in the promotion of gene expression essential for osteogenesis, such as osteocalcin and bone sialoprotein [23]. Activation of PERK leads to the phosphorylation of the α-subunit of the eukaryotic initiation factor 2 (eIF2), which reduces protein translation in response to ER stress and also osteoclastogenesis [24], [25]. Deficiency of PERK in mice leads to severe neonatal osteopenia associated with impairment of osteoblast proliferation and differentiation and of trafficking of type I procollagen [26]. The fact that F decreased the abundance of PERK when compared to the control A/J group, may indicate both negative and positive regulation of F in osteoblast differentiation and osteoclastogenesis in this strain, respectively. No differences in PERK abundance were observed when comparing other groups.

The treatment with high levels of F increased expression of SHIP1/2 in both A/J and 129P3/J mice. SHIP dephosphorylates the 5′-phosphate group from phosphatidylinositol-3,4,5-triphosphate (PIP3), the major product of phosphatidylinositol 3-kinase (PI3-K), inactivating it. This signaling is implicated in macrophage-colony stimulating factor and receptor activator of nuclear factor-κB ligand-mediated osteoclast activation [27]. SHIP-/- mice showed an increase in numbers of osteoclast precursors in bone marrow, and the number of osteoclasts was also increased two-fold in bone [28]. The enhancement of SHIP1 abundance in both strains after F suggests that it down regulates osteoclast differentiation, regardless the strain.

Another identified protein involved in bone resorption and osteoclast regulation with differences in abundance is Nox4. NADPH oxidase is an enzyme system responsible for producing osteoclastic superoxide radicals, promoting bone resorption [29]. Superoxides may be involved in activation of the transcription factor NF-kB, which enhances the transcription of genes signaling osteoclastic activation [30]. This enzyme was found to be more abundant in the control 129P3/J compared to control A/J mice and was modulated by both levels of F in that strain. However, F at only 10 ppm increased its abundance in bone of A/J mice but not in 129P3/J mice. The differential increase in abundance of Nox4 after F treatment in both strains may suggest that it regulates bone remodeling by enhancing the osteoclast activity in both strain-specific and dose-specific manners.

Detoxification enzymes, such as AFB1-AR and CBR, showed an increase in abundance after 50 ppmF treatment in 129P3/J mice compared to the control group. The CBR was identified in mice lung tissue and may have a role in the detoxification of xenobiotics and of toxic aldehydes derived from lipid peroxidation processes [31]. AFB1-AR, in turn, protects the liver against the toxic effect of aflatoxin B1 (AFB1) [32]. F has been shown to induce lipid peroxidation, as a dose-dependent toxic effect [33]. Thus, it is possible that F at high doses induces lipid peroxidation in bone cells, as judged by increased levels of CBR and AFB1-AR. This suggests that bone cells from 129P3/J but not from A/J mice require molecular mechanisms to avoid the F-induced toxicity. Recently, it was suggested that many species have F riboswitches to control the expression of proteins that soften the deleterious effects of this anion [34]. It is possible that cells from the A/J and 129P3/J strains differ in the abundance and/or usage of those F-sensing RNAs.

These results suggest that F targets molecular mechanisms in both osteoblasts and osteoclasts. This is consistent with the fact that F acts on osteoblasts and osteoclasts *in vivo* and *in vitro*, by still uncertain mechanisms [5]. F promotes both osteoblast proliferation and death at low and high concentrations, respectively [5], [7]. Moreover, the genetic background constitutes an important factor for the differential effect of F on osteoclasts [35], [36].

**Figure 6:**
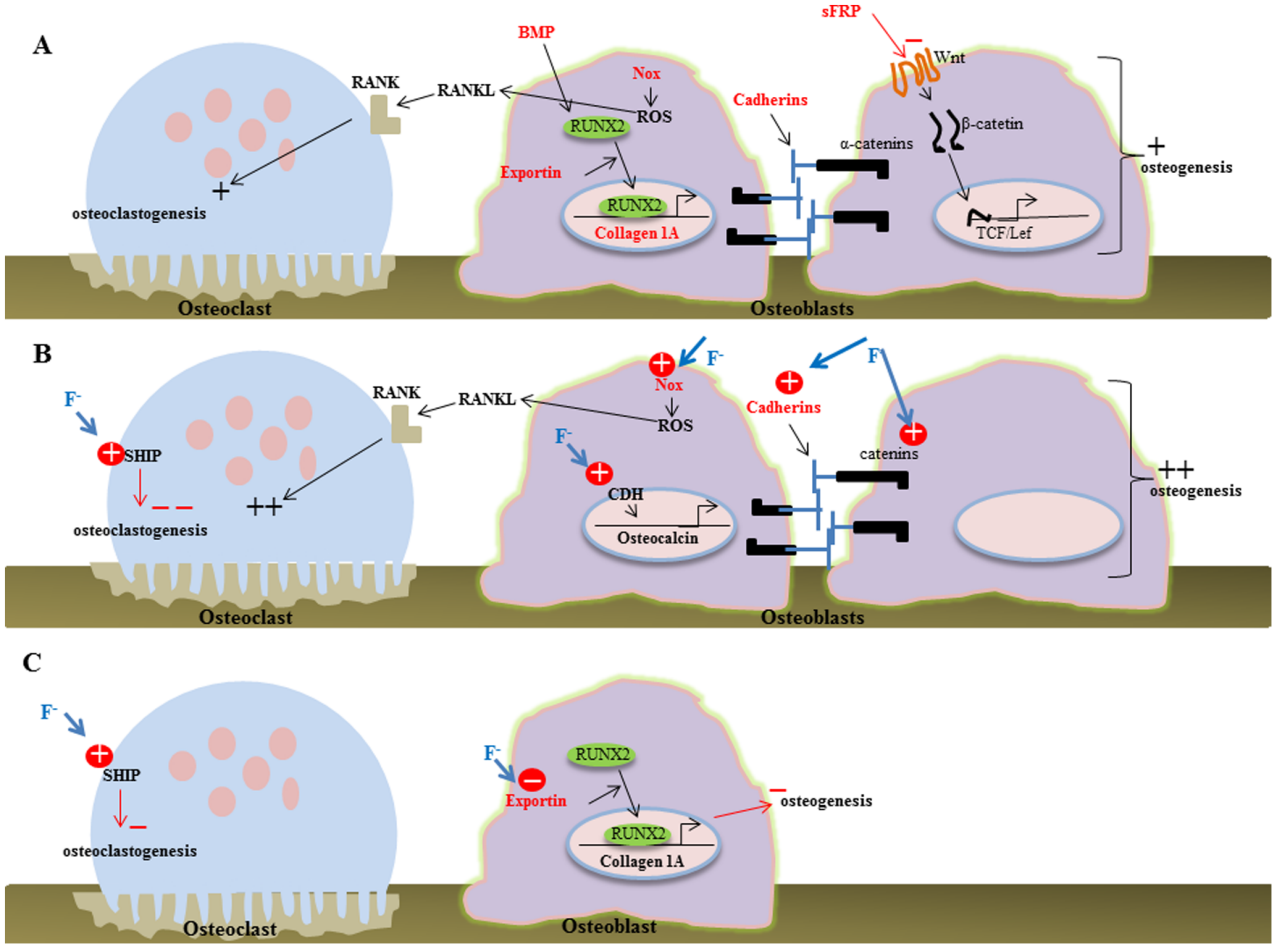
Schemes of the protein-based mechanisms triggered by F in osteoblasts and osteoclasts. Identified proteins with greater abundance in 129P3/J in comparison to A/J related to bone metabolism are shown in red. (A) Exportin enhances transport of cfab/Runx-2 to the nucleus activating transcription of collagen type I genes. Cadherins promote intercellular adhesion enhancing osteoblast differentiation. Nox-mediated ROS production enhances RANK ligand (RANKL) that interacts to its receptor (RANK) to induce osteoclast differentiation. (B) The treatment of 129P3/J with high F promotes enhancement of Nox, CDH, cadherin, catetin and SHIP while (C) in A/J, F at high dose promotes enhancement of SHIP and reduction of exportin.

Taken together, 129P3/J and A/J mice have intrinsic molecular differences related to bone metabolism (Fig. 6*A*). They also differ in response to F. 129P3/J mice responded similarly at high and low F doses, increasing the level of proteins involved in bone formation, as well as in bone resorption (Fig. 6*B*), a similar profile observed for low F-treated A/J mice. Instead, high F treatment diminishes level of proteins related to bone remodeling (Fig. 6*C*). These findings are consistent with known differences in susceptibility to F effect on mineralized tissues between these strains. The A/J mouse strain is more sensitive to develop dental fluorosis and to alterations in the quality of bone, while 129P3/J is less affected. Proteomic data revealed that bones of A/J mice were highly responsive to F which altered the abundance of 36 proteins even at the low dose level. At the higher F exposure level, A/J mice had 17 proteins with differences in expression compared to the control (untreated) mice. On the other hand, the treatment of 129P3/J mice with low and high doses of F altered 29 and 127 proteins, respectively. The greater responsiveness to high dose of F suggests that, possibly due to its resistance, 129P3/J requires a higher dose to trigger cytotoxic, detox and anabolic responses. Thus, at the higher dose F can stimulate bone formation in 129P3/J mice and either not change or decrease it in A/J mice.

One of the major obstacles of osteoporosis therapy with F is the difficulty in prospectively identifying which patients might benefit from therapy, since not all patients respond to F treatment [7]. In the present study, F exposure led to specific changes in the expression of proteins in both strains evaluated, showing that there is an influence of genetic background in bone cell responses to F. Based on its greater F-mediated bone formation, we found the 129P3/J strain to be a good responder to F. Additional studies should identify, in humans, biomarkers of bone tissue response to F, with the aim of identifying the best candidates to receive F therapy in diseases such as osteoporosis.

## Supporting Information

S1 Figure BMD and histomorphometric parameters in trabecular region of 4^th^ lumbar vertebrae from A/J and 129P3/J mice treated with 0, 10 or 50 ppm F. Values are mean ± SD, n = 8/group. A =  Bone mineral density (BMD); B =  Specific bone surface BS/BV; C =  Bone volume fraction (BV/TV); D =  Bone surface density (BS/TV); E =  Trabecular number (Tb.N); F =  Trabecular thickness (Tb.Th); G =  Trabecular separation (Tb.Sp); H =  Trabecular bone pattern factor (Tb.Pf). *p<0.05 and **p<0.01 represent significant differences between strains, for each group.
(DOCX)

S2 Figure Biological process distribution of the identified bone proteins with differences in abundance among F treatments in A/J and 129P3/J mice, n = 8/group.
(DOCX)

S3 Figure Cellular distribution of the identified bone proteins with differences in abundance among F treatments in A/J and 129P3/J mice, n = 8/group.
(DOCX)

S4 Figure Biological process distribution of the identified bone proteins with differences in abundance between the strains (A/J and 129P3/J) for each F treatment, n = 8/group.
(DOCX)

S5 Figure Cellular distribution of the identified bone proteins with differences in abundance between the strains (A/J and 129P3/J) for each F treatment, n = 8/group.
(DOCX)

S1 Table Complete list of identified proteins with differences in abundance in the comparison between control 129P3/J and 10 ppmF-treated 129P3/J mice.
(DOCX)

S2 Table Complete list of identified proteins with differences in abundance in the comparison between control 129P3/J and 50 ppmF-treated 129P3/J mice.
(DOCX)

S3 Table Complete list of identified proteins with differences in abundance in the comparison between 10 ppmF-treated 129P3/J and 50 ppmF-treated 129P3/J mice.
(DOCX)

S4 Table Complete list of identified proteins with differences in abundance in the comparison between control A/J and 10 ppmF– treated mice.
(DOCX)

S5 Table Complete list of identified proteins with differences in abundance in the comparison between control A/J and 50 ppmF-treated A/J mice.
(DOCX)

S6 Table Complete list of identified proteins with differences in abundance in the comparison between 10 ppmF-treated A/J and 50 ppmF-treated A/J mice.
(DOCX)

S7 Table Complete list of identified proteins with differences in abundance in the comparison between control 129P3/J and control A/J mice.
(DOCX)

S8 Table Complete list of identified proteins with differences in abundance in the comparison between 10 ppmF-treated 129P3/J and 10 ppmF-treated A/J mice.
(DOCX)

S9 Table Complete list of identified proteins with differences in abundance in the comparison between 50 ppmF-treated 129P3/J and 50 ppmF-treated A/J mice.
(DOCX)
