# Supplementary material for: Bone Response to Fluoride Exposure Is Influenced by Genetics
Source: PLoS One. 2014 Dec 11;9(12):e114343. doi: 10.1371/journal.pone.0114343 (PMC4263599; doi:10.1371/journal.pone.0114343)
Supplement: S1 Figure — BMD and histomorphometric parameters in trabecular region of 4th lumbar vertebrae from A/J and 129P3/J mice treated with 0, 10 or 50 ppm F. Values are mean ± SD, n = 8/group. A = Bone mineral density (BMD); B = Specific bone surface BS/BV; C = Bone volume fraction (BV/TV); D = Bone surface density (BS/TV); E = Trabecular number (Tb.N); F = Trabecular thickness (Tb.Th); G = Trabecular separation (Tb.Sp); H = Trabecular bone pattern factor (Tb.Pf). *p<0.05 and **p<0.01 represent significant differences between strains, for each group. (DOCX) [file pone.0114343.s001.docx]

**Supplemental Figure 1**
