# Supplementary figures and images for: Bone Response to Fluoride Exposure Is Influenced by Genetics
Source: PLoS One. 2014 Dec 11;9(12):e114343. doi: 10.1371/journal.pone.0114343 (PMC4263599; doi:10.1371/journal.pone.0114343)

**Supplemental Figure 2**

**
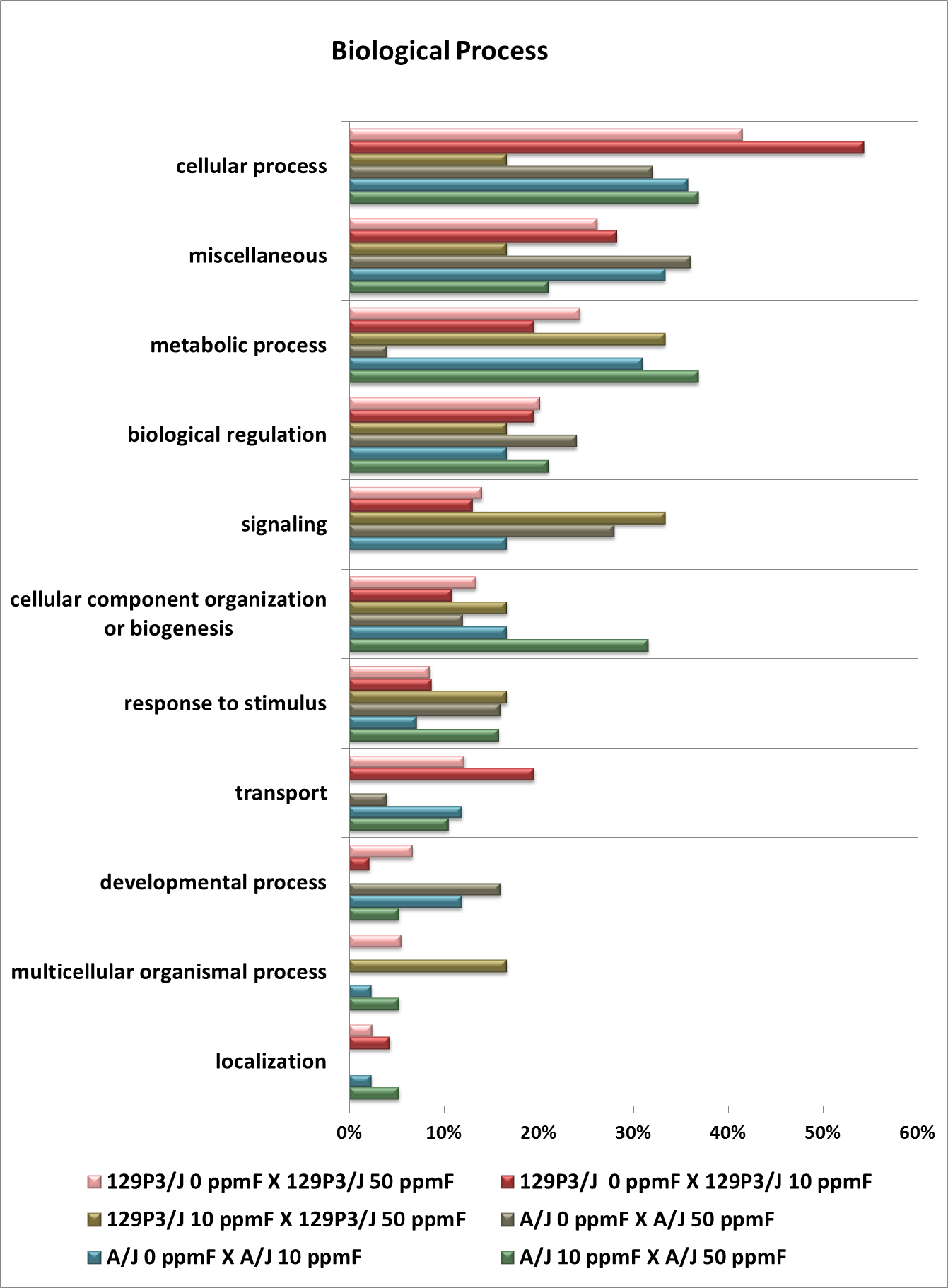
**

Supplement: S2 Figure — Biological process distribution of the identified bone proteins with differences in abundance among F treatments in A/J and 129P3/J mice, n = 8/group. (DOCX) [file pone.0114343.s002.docx]

**Supplemental Figure
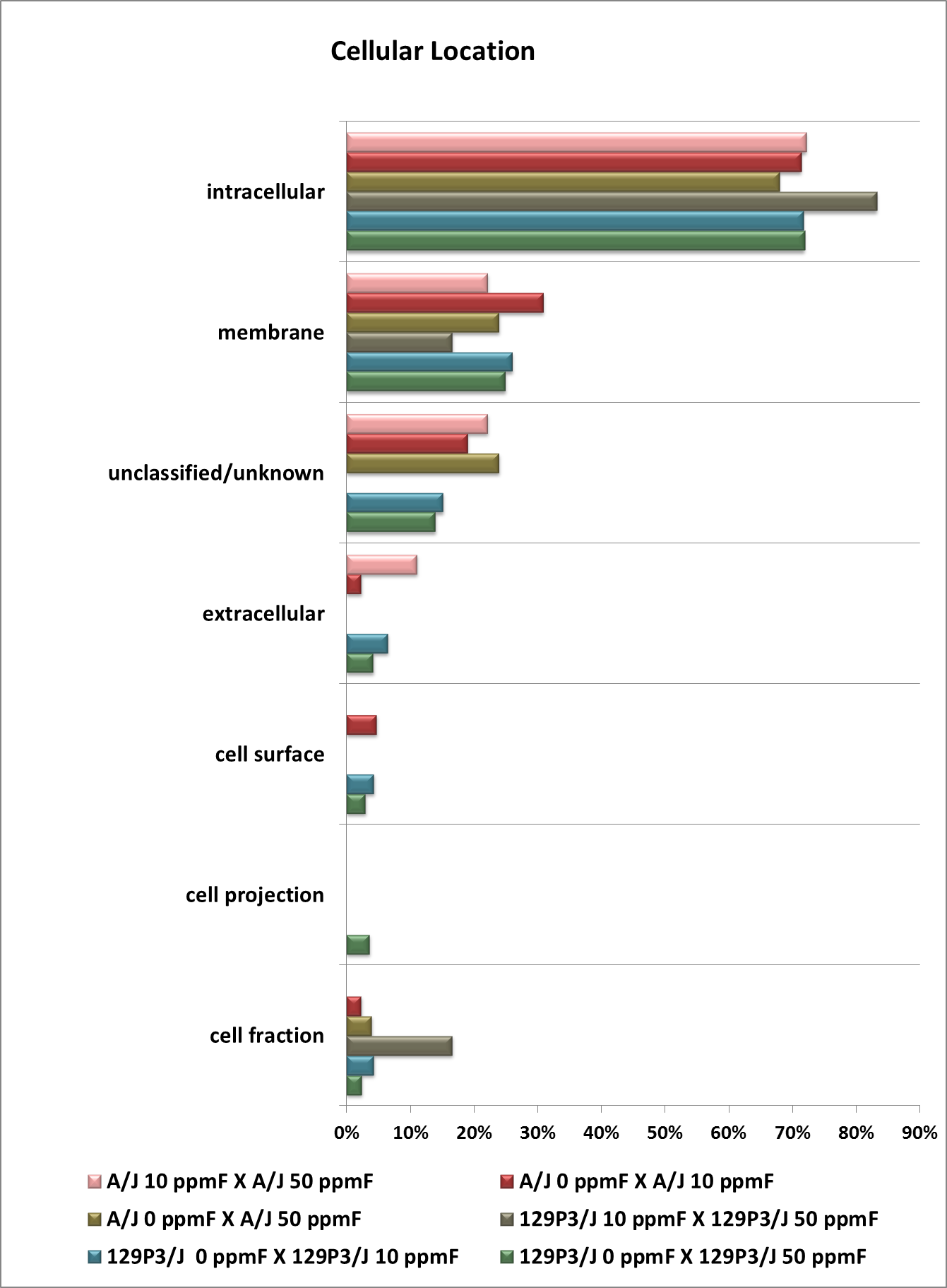
3**

Supplement: S3 Figure — Cellular distribution of the identified bone proteins with differences in abundance among F treatments in A/J and 129P3/J mice, n = 8/group. (DOCX) [file pone.0114343.s003.docx]

**Supplemental Figure
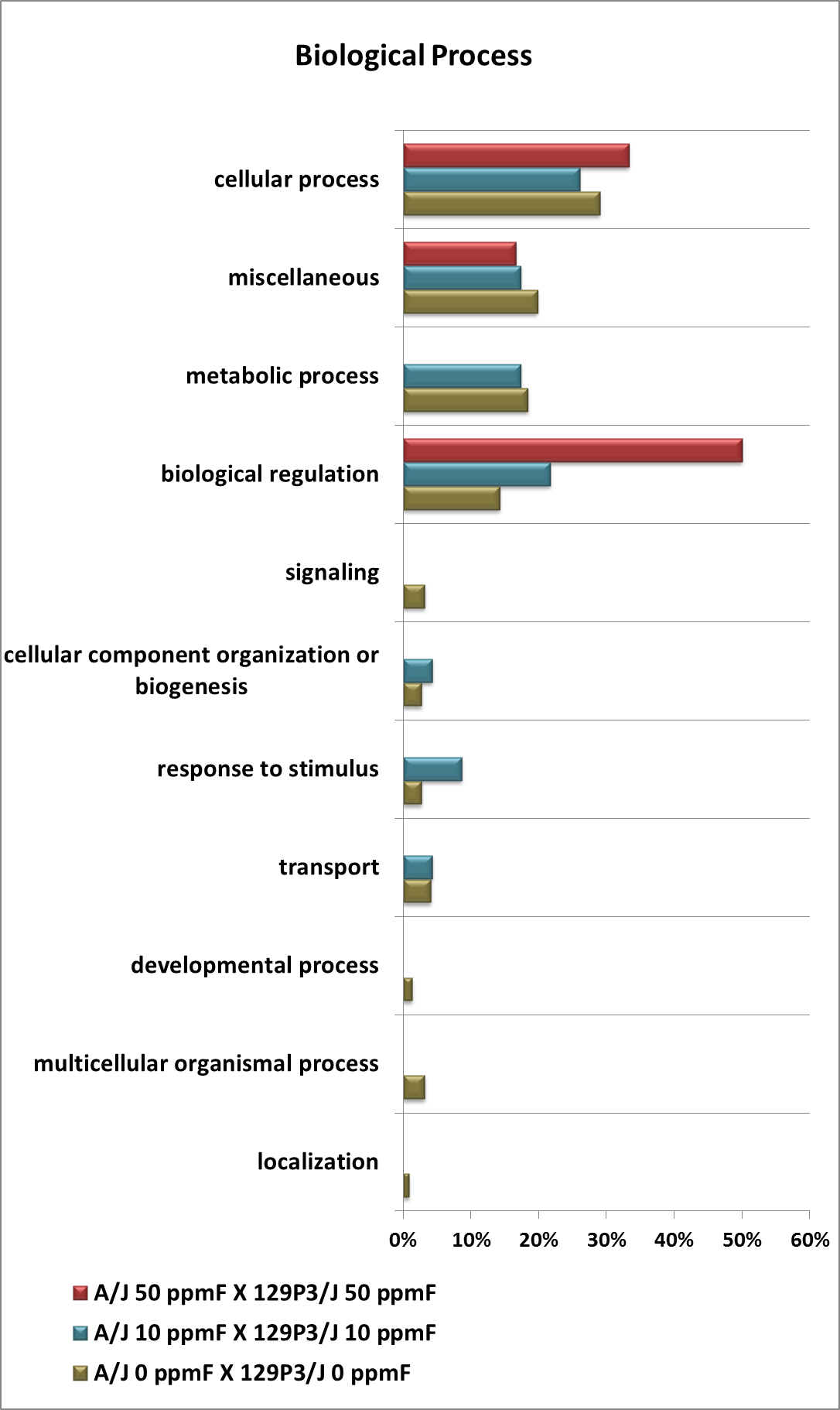
4**

Supplement: S4 Figure — Biological process distribution of the identified bone proteins with differences in abundance between the strains (A/J and 129P3/J) for each F treatment, n = 8/group. (DOCX) [file pone.0114343.s004.docx]

**Supplemental Figure 5**

**
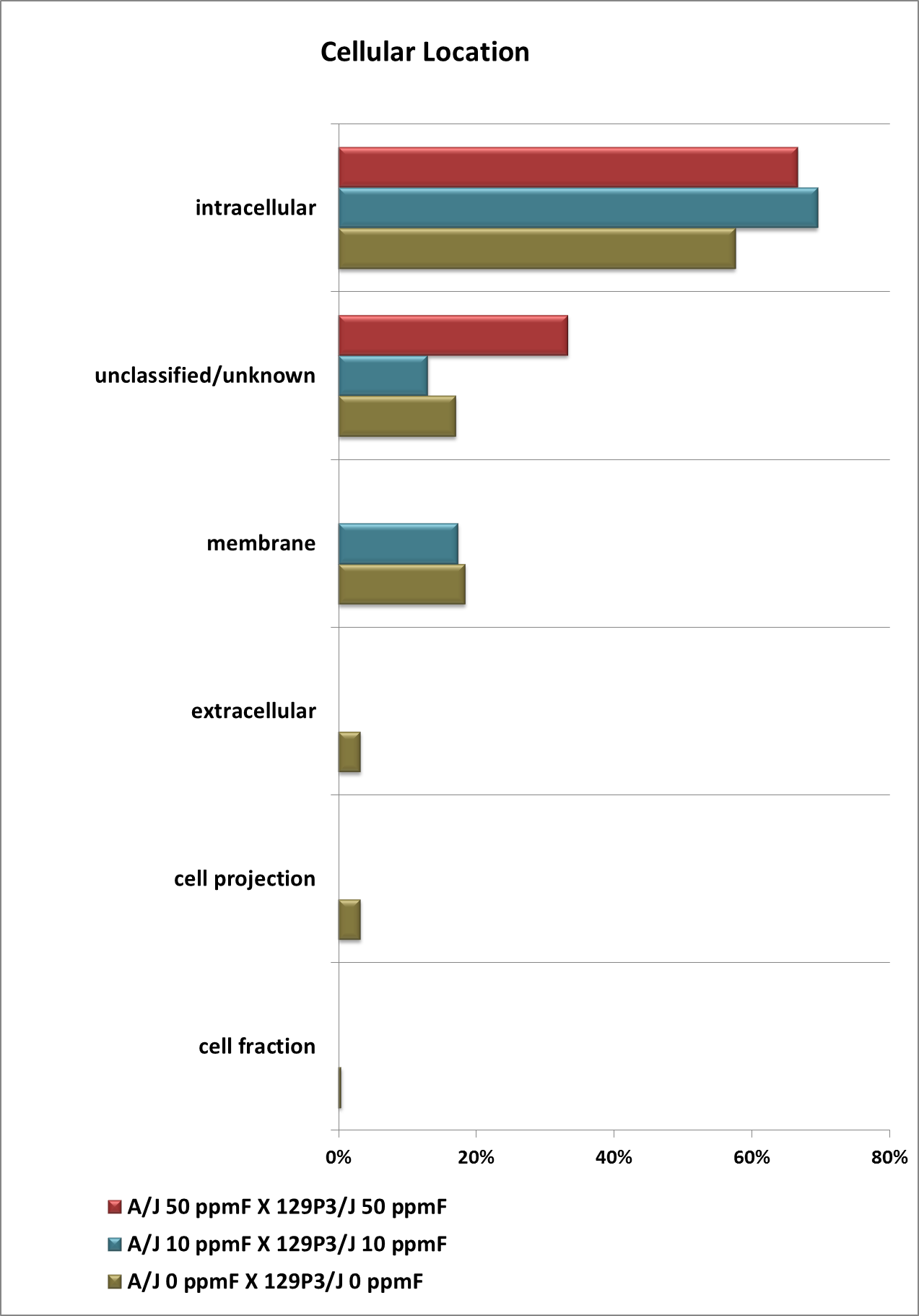
**

Supplement: S5 Figure — Cellular distribution of the identified bone proteins with differences in abundance between the strains (A/J and 129P3/J) for each F treatment, n = 8/group. (DOCX) [file pone.0114343.s005.docx]
