# Supplementary material for: Bone Response to Fluoride Exposure Is Influenced by Genetics
Source: PLoS One. 2014 Dec 11;9(12):e114343. doi: 10.1371/journal.pone.0114343 (PMC4263599; doi:10.1371/journal.pone.0114343)
Supplement: S1 Table — Complete list of identified proteins with differences in abundance in the comparison between control 129P3/J and 10 ppmF-treated 129P3/J mice. (DOCX) [file pone.0114343.s006.docx]

**Supplemental Table 1.** Identified proteins with differences in abundance in the comparison between control 129P3/J and 10 ppmF-treated 129P3/J mice.

| **Acession Number*^a^*** | **Protein*^b^*** | **Ratio*^c^*** | **Nº of peptides*^d^*** |
| --- | --- | --- | --- |
| E9Q6Y8 | Ubiquitin carboxyl-terminal hydrolase | 0.5 | 3 |
| Q80Z37 | E3 ubiquitin-protein ligase Topors | 0.5 | 2 |
| Q8K341 | Alpha-tubulin N-acetyltransferase | 0.5 | 2 |
| Q3UHE1 | Membrane-associated phosphatidylinositol transfer protein 3 | 0.5 | 2 |
| Q9Z0X4 | cGMP-inhibited 3',5'-cyclic phosphodiesterase A | 0.5 | 2 |
| Q9QXS1 | Plectin | 0.5 | 2 |
| Q8R3E3 | WD repeat domain phosphoinositide-interacting protein 1 | 0.5 | 2 |
| E9Q5L8 | Protein Glyatl3 | 0.5 | 2 |
| Q8BVE8 | Histone-lysine N-methyltransferase NSD2 | 0.5 | 2 |
| Q8C419 | Probable G-protein coupled receptor 158 | 0.5 | 2 |
| Q8BIY1 | G patch domain-containing protein 3 | 0.5 | 2 |
| Q9Z1J3 | Cysteine desulfurase, mitochondrial | 0.5 | 2 |
| B2RXE2 | Sodium/hydrogen exchanger | 0.5 | 2 |
| Q8BPY9 | Fidgetin-like protein 1 | 0.5 | 2 |
| Q99N50 | Synaptotagmin-like protein 2 | 0.5 | 2 |
| Q8BMJ2 | Leucine--tRNA ligase, cytoplasmic | 0.5 | 2 |
| Q9DA19 | Corepressor interacting with RBPJ 1 | 0.5 | 2 |
| Q3UEG8 | Complement factor B, isoform CRA_a | 0.5 | 2 |
| B8JJM6 | Complement factor B | 0.5 | 2 |
| Q2EMV9 | Poly [ADP-ribose] polymerase 14 | 0.5 | 2 |
| Q3TAP4 | AP-5 complex subunit beta-1 | 0.5 | 2 |
| Q6QIY3 | Sodium channel protein type 10 subunit alpha | 0.5 | 2 |
| P58545 | BTB/POZ domain-containing protein 3 | 0.5 | 2 |
| Q9JHI8 | NADPH oxidase 4 | 0.5 | 2 |
| Q0VBM2 | Protein FAM83B | 0.5 | 2 |
| Q9DBG7 | Signal recognition particle receptor subunit alpha | 0.4 | 2 |
| Q8CIN4 | Serine/threonine-protein kinase PAK 2 | 0.4 | 2 |
| A2ASQ1 | agrin | 0.4 | 2 |
| Q569Z6 | Thyroid hormone receptor-associated protein 3 | 0.4 | 2 |

*^a^*Protein accession numbers from UniProtKB. *^b^*Protein name. *^c^*Ratio of the relative protein abundance between (A) control 129P3/J and (B) 10 ppmF-treated 129P3/J mice. Significant differences in protein abundance were considered when ratio ≤ 0.5 or ≥ 1.5. Ratio ≤ 0.5 means increase in group B in relation to group A and ratio ≥ 1.5 means decrease in group B in relation to group A. *^d^*Number of peptides identified.
