# Supplementary material for: Bone Response to Fluoride Exposure Is Influenced by Genetics
Source: PLoS One. 2014 Dec 11;9(12):e114343. doi: 10.1371/journal.pone.0114343 (PMC4263599; doi:10.1371/journal.pone.0114343)
Supplement: S2 Table — Complete list of identified proteins with differences in abundance in the comparison between control 129P3/J and 50 ppmF-treated 129P3/J mice. (DOCX) [file pone.0114343.s007.docx]

**Supplemental Table 2.** Identified proteins with differences in abundance in the comparison between control 129P3/J and 50 ppmF-treated 129P3/J mice.

| **Acession Number*^a^*** | **Protein*^b^*** | **Ratio*^c^*** | **Nº of peptides*^d^*** |
| --- | --- | --- | --- |
| Q0P5X5 | zinc finger B_box domain_containing protein 1 | 1.8 | 2 |
| Q3TY86 | apoptosis_inducing factor 3 | 0.5 | 4 |
| [E9QPK4](http://www.uniprot.org/uniprot/E9QPK4) | serine/threonine_protein kinase ATR | 0.5 | 2 |
| [Q60865](http://www.uniprot.org/uniprot/Q60865) | caprin_1 | 0.5 | 2 |
| [Q9WTS2](http://www.uniprot.org/uniprot/Q9WTS2) | alpha_(1_6)_fucosyltransferase | 0.5 | 2 |
| [Q6QIY3](http://www.uniprot.org/uniprot/Q6QIY3) | sodium channel protein type 10 subunit alpha | 0.5 | 3 |
| Q9CX56 | 26S proteasome non_ATPase regulatory subunit 8 | 0.5 | 2 |
| [Q9QZZ4](http://www.uniprot.org/uniprot/Q9QZZ4) | myosin_XV | 0.5 | 5 |
| O08810 | 116 kDa U5 small nuclear ribonucleoprotein component | 0.5 | 2 |
| [A2A5N8](http://www.uniprot.org/uniprot/A2A5N8) | lethal(3)malignant brain tumor_like protein | 0.5 | 2 |
| P70399 | tumor suppressor p53_binding protein 1 | 0.5 | 3 |
| [Q8CG76](http://www.uniprot.org/uniprot/Q8CG76) | aflatoxin B1 aldehyde reductase member 2 | 0.5 | 2 |
| [Q05KA5](http://www.uniprot.org/uniprot/Q05KA5) | Submandibular androgen-repressed protein | 0.5 | 2 |
| Q99K41 | EMILIN-1 | 0.5 | 4 |
| Q60665 | Ski-like protein | 0.5 | 2 |
| P33610 | DNA primase large subunit | 0.5 | 2 |
| Q9CUU3 | Synaptonemal complex protein 2 | 0.5 | 4 |
| E9Q796 | Protein Scml2 | 0.5 | 3 |
| Q8R4P4 | Transmembrane channel-like protein 2 | 0.5 | 2 |
| Q9CSB4 | Partitioning defective 3 homolog B | 0.5 | 2 |
| Q8BGD9 | Eukaryotic translation initiation factor 4B | 0.5 | 2 |
| B2RXE2 | Sodium/hydrogen exchanger | 0.5 | 2 |
| P0C7T6 | Ataxin-1-like | 0.5 | 2 |
| Q6ZPI0 | Protein Jade-1 | 0.5 | 2 |
| Q91XZ1 | Protocadherin beta 9 | 0.5 | 2 |
| Q9CY66 | H/ACA ribonucleoprotein complex subunit 1 | 0.5 | 3 |
| Q91V87 | Fibroblast growth factor receptor-like 1 | 0.5 | 2 |
| O09053 | Werner syndrome ATP-dependent helicase homolog | 0.5 | 2 |
| Q09LZ8 | Cytosolic carboxypeptidase 6 | 0.5 | 2 |
| F2Z4A3 | Protein Fat1 | 0.5 | 2 |
| Q8CJ19 | Protein-methionine sulfoxide oxidase MICAL3 | 0.5 | 2 |
| A2A3V1 | A-kinase anchor protein 17B | 0.5 | 2 |
| P43277 | Histone H1.3 | 0.5 | 2 |
| P15864 | Histone H1.2 | 0.5 | 2 |
| Q3UHC2 | Leucine-rich repeat serine/threonine-protein kinase 1 | 0.5 | 2 |
| Q8VHE6 | Dynein heavy chain 5, axonemal | 0.5 | 2 |
| Q60519 | Semaphorin-5B | 0.5 | 2 |
| Q9WTV7 | E3 ubiquitin-protein ligase RLIM | 0.5 | 2 |
| Q9QXS1 | Plectin | 0.5 | 2 |
| Q9WV02 | RNA-binding motif protein, X chromosome | 0.5 | 3 |
| Q91VM5 | RNA binding motif protein, X-linked-like-1 | 0.5 | 3 |
| Q8CHP0 | Zinc finger CCCH domain-containing protein 3 | 0.5 | 2 |
| Q75N62 | GTPase IMAP family member 8 | 0.5 | 3 |
| E9Q6J5 | Protein Bod1l | 0.5 | 2 |
| A3KMG9 | Npc1l1 protein | 0.5 | 2 |
| Q8CBF3 | Ephrin type-B receptor 1 | 0.5 | 3 |
| Q9CRA2 | PDZ and LIM domain protein 5 | 0.5 | 2 |
| Q91VY9 | Zinc finger protein 622 | 0.5 | 2 |
| Q8BI79 | Coiled-coil domain-containing protein 40 | 0.5 | 2 |
| P08074 | Carbonyl reductase [NADPH] 2 | 0.5 | 2 |
| G5E8L9 | Mroh9 | 0.5 | 2 |
| Q69ZK0 | Phosphatidylinositol 3,4,5-trisphosphate-dependent Rac exchanger 1 protein | 0.5 | 2 |
| Q7TQG0 | Zinc finger and BTB domain-containing protein 5 | 0.5 | 2 |
| Q673H1 | Tumor suppressor candidate gene 1 protein homolog | 0.5 | 3 |
| Q6AXB7 | Fragile X mental retardation protein 1 homolog | 0.5 | 3 |
| Q8JZL7 | Ras-GEF domain-containing family member 1B | 0.5 | 2 |
| Q6VNB8 | WD repeat and FYVE domain-containing protein 3 | 0.5 | 2 |
| [A2AJK6](http://www.uniprot.org/uniprot/A2AJK6) | Chromodomain-helicase-DNA-binding protein 7 | 0.5 | 2 |
| Q5DTV4 | Probable ribonuclease ZC3H12C | 0.5 | 2 |
| Q67FY2 | B-cell CLL/lymphoma 9-like protein | 0.5 | 2 |
| Q8BMI0 | F-box only protein 38 | 0.5 | 3 |
| Q9JJA9 | General receptor for phosphoinositides 1-associated scaffold protein | 0.5 | 2 |
| Q6PDM4 | Coiled-coil domain-containing protein 36 | 0.5 | 2 |
| Q99N48 | Synaptotagmin-like protein 3 | 0.5 | 2 |
| E9Q6B1 | Protein Cylc2 | 0.5 | 2 |
| Q80TE4 | Signal-induced proliferation-associated 1-like protein 2 | 0.5 | 2 |
| Q91VW5 | Golgin subfamily A member 4 | 0.5 | 2 |
| Q9JJN5 | Carboxypeptidase N catalytic chain | 0.5 | 2 |
| O09106 | Histone deacetylase 1 | 0.5 | 2 |
| P70288 | Histone deacetylase 2 | 0.5 | 2 |
| Q8C0F9 | Inactive serine protease 35 | 0.5 | 2 |
| Q0VGY8 | Protein TANC1 | 0.4 | 3 |
| Q8BIY1 | G patch domain-containing protein 3 | 0.4 | 2 |
| Q8CIH5 | 1-phosphatidylinositol 4,5-bisphosphate phosphodiesterase gamma-2 | 0.4 | 3 |
| A2AGT5 | Cytoskeleton-associated protein 5 | 0.4 | 2 |
| Q9Z0X4 | cGMP-inhibited 3',5'-cyclic phosphodiesterase A | 0.4 | 2 |
| Q8C8K6 | Ataxin 7-like 2 | 0.4 | 2 |
| Q8K1N2 | Pleckstrin homology-like domain family B member 2 | 0.4 | 2 |
| Q8BMA3 | Connector enhancer of kinase suppressor of ras 3 | 0.4 | 2 |
| F8WIA8 | MAGE-like protein 2 | 0.4 | 3 |
| Q5STT6 | Protein FAM71B | 0.4 | 2 |
| Q8BZ47 | Zinc finger protein 609 | 0.4 | 2 |
| E9QLJ0 | Cardiomyopathy-associated protein 5 | 0.4 | 3 |
| E9Q414 | Apolipoprotein B-100 | 0.4 | 3 |
| Q91XQ0 | Dynein heavy chain 8, axonemal | 0.4 | 3 |
| E9Q5F9 | Histone-lysine N-methyltransferase SETD2 | 0.4 | 3 |
| E9Q774 | Protein Akap11 | 0.4 | 2 |
| Q6P549 | Phosphatidylinositol 3,4,5-trisphosphate 5-phosphatase 2 | 0.4 | 3 |
| P97855 | Ras GTPase-activating protein-binding protein 1 | 0.4 | 2 |
| Q8BWJ3 | Phosphorylase b kinase regulatory subunit alpha, liver isoform | 0.4 | 2 |
| P70670 | Nascent polypeptide-associated complex subunit alpha, muscle-specific form | 0.4 | 3 |
| A2AMT1 | Filensin | 0.4 | 2 |
| P47911 | 60S ribosomal protein L6 | 0.4 | 2 |
| Q8CIN4 | Serine/threonine-protein kinase PAK 2 | 0.4 | 3 |
| Q8K341 | Alpha-tubulin N-acetyltransferase | 0.4 | 2 |
| [A2ASQ1](http://www.uniprot.org/uniprot/A2ASQ1) | Agrin | 0.4 | 2 |
| P35487 | Pyruvate dehydrogenase E1 component subunit alpha, testis-specific form, mitochondrial | 0.4 | 2 |
| Q6PDQ2 | Chromodomain-helicase-DNA-binding protein 4 | 0.4 | 4 |
| [Q7TNV0](http://www.uniprot.org/uniprot/Q7TNV0) | protein DEK | 0.4 | 2 |
| Q8C8K3 | Serine/arginine-rich splicing factor 12 | 0.4 | 2 |
| Q99N50 | Synaptotagmin-like protein 2 | 0.4 | 2 |
| Q641K1 | cytosolic carboxypeptidase 1 | 0.4 | 2 |
| B8JJEO | G-protein coupled receptor 98 | 0.4 | 3 |
| Q99PI5 | phosphatidate phosphatase LPIN2 | 0.4 | 2 |
| Q9WTU0 | Lysine-specific demethylase PHF2 | 0.4 | 3 |
| Q9DA19 | Corepressor interacting with RBPJ 1 | 0.3 | 2 |
| O35348 | Acetylcholinesterase collagenic tail peptide | 0.3 | 2 |
| Q7TNF8 | Peripheral-type benzodiazepine receptor-associated protein 1 | 0.3 | 2 |
| P58545 | BTB/POZ domain-containing protein 3 | 0.3 | 2 |
| Q7TT79 | Microcephalin | 0.3 | 2 |
| Q7TQK0 | cyclin T2 | 0.3 | 2 |
| Q9WTL4 | Insulin receptor-related protein precursor | 0.3 | 2 |
| Q0GNC1 | Inverted formin-2 | 0.3 | 2 |
| Q3TAP4 | AP-5 complex subunit beta-1 | 0.3 | 2 |
| Q0VBM2 | Protein FAM83B | 0.3 | 2 |
| Q9Z2Q2 | Lysine-richnucleolar protein 1 | 0.3 | 3 |
| Q80YA3 | Phospholipase DDHD1 | 0.3 | 2 |
| Q10470 | Beta-1,4-mannosyl-glycoprotein 4-beta-N-Acetylglucosaminyltransferase | 0.3 | 2 |
| Q8BMJ2 | Leucine-tRNA ligase, cytoplasmic | 0.3 | 2 |
| Q4QRL3 | Coiled_coil domain_containing protein 88B | 0.3 | 2 |
| Q8BL06 | Inactive ubiquitin carboxyl-terminal hydrolase | 0.3 | 3 |
| Q3U2J5 | Calmodulin-lysine N-methyltransferase | 0.3 | 2 |
| Q9D3E6 | Cohesin subunit SA-1 | 0.2 | 2 |
| Q61301 | Catenin alpha-2 | 0.2 | 2 |
| Q9JHI8 | NADPH oxidase 4 | 0.2 | 2 |
| Q8K394 | Inactive phospholipase C-like protein 2 | 0.2 | 2 |
| Q569Z6 | Thyroid hormone receptor-associated protein 3 | 0.2 | 2 |

*^a^*Protein accession numbers from UniProtKB. *^b^*Protein name. *^c^*Ratio of the relative protein abundance between (A) control 129P3/J and (B) 50 ppmF-treated 129P3/J mice. Significant differences in protein abundance were considered when ratio ≤ 0.5 or ≥ 1.5. Ratio ≤ 0.5 means increase in group B in relation to group A and ratio ≥ 1.5 means decrease in group B in relation to group A. *^d^*Number of peptides identified.
