# Supplementary material for: Bone Response to Fluoride Exposure Is Influenced by Genetics
Source: PLoS One. 2014 Dec 11;9(12):e114343. doi: 10.1371/journal.pone.0114343 (PMC4263599; doi:10.1371/journal.pone.0114343)
Supplement: S4 Table — Complete list of identified proteins with differences in abundance in the comparison between control A/J and 10 ppmF– treated mice. (DOCX) [file pone.0114343.s009.docx]

**Supplemental Table 4.** Identified proteins with differences in abundance in the comparison between control A/J and 10 ppmF– treated mice.

| Acession Number*^a^* | Protein*^b^* | Ratio*^c^* | Nº of peptides*^d^* |
| --- | --- | --- | --- |
| Q8R087 | Beta-1-4-galactosyltransferase 7 | 1.6 | 2 |
| Q8R4P4 | transmembrane channel-like protein 2 | 0.5 | 2 |
| Q8BUH8 | Sentrin-specific protease 7 | 0.5 | 2 |
| E9Q0Y4 | Signal-induced proliferation-associated protein 1 | 0.5 | 2 |
| Q9ERK4 | Exportin-2 | 0.5 | 2 |
| A2ASQ1 | agrin | 0.5 | 2 |
| Q8K0D5 | elongation factor G- mitochondrial precursor | 0.5 | 2 |
| E9Q9M9 | kelch domain containing 7B | 0.5 | 2 |
| Q5RKT9 | Beta-1-4-mannosyl-glycoprotein 4-beta-N-acetylglucosaminyltransferase | 0.5 | 2 |
| Q8C9B9 | Death-inducer obliterator 1 | 0.5 | 2 |
| Q8K341 | Alpha-tubulin N-acetyltransferase | 0.5 | 2 |
| Q9Z0X4 | cGMP-inhibited 3',5'-cyclic phosphodiesterase A | 0.5 | 2 |
| A2ARJ3 | Transmembrane protein 236 | 0.5 | 2 |
| Q80XK6 | Autophagy-related protein 2 homolog B | 0.5 | 2 |
| Q0VAV5 | RAS protein activator like 2 | 0.5 | 3 |
| Q7TSU7 | Kin of IRRE-like protein 2 | 0.5 | 2 |
| E9PY16 | Protein Adap1 centaurin- alpha 1 | 0.5 | 2 |
| G3X928 | SEC23-interacting protein | 0.5 | 2 |
| Q3V3R1 | Monofunctional C1-tetrahydrofolate synthase, mitochondrial | 0.5 | 2 |
| B2RQE8 | Rho GTPase-activating protein 42 | 0.5 | 2 |
| Q0KK59 | Protein unc-79 homolog | 0.4 | 2 |
| Q4QY64 | ATPase family AAA domain-containing protein 5 | 0.4 | 4 |
| Q91XQ0 | Dynein heavy chain 8, axonemal | 0.4 | 2 |
| Q9WTU0 | Lysine-specific demethylase PHF2 | 0.4 | 3 |
| A2AJK6 | Chromodomain-helicase-DNA-binding protein 7 | 0.4 | 2 |
| Q0GNC1 | Inverted formin-2 | 0.4 | 2 |
| Q6ZWQ0 | Nesprin-2 | 0.4 | 2 |
| Q9JHI8 | NADPH oxidase 4 | 0.4 | 2 |
| Q8BTI8 | Serine/arginine repetitive matrix protein 2 | 0.4 | 4 |
| Q9D3E6 | Cohesin subunit SA-1 | 0.3 | 2 |
| P35487 | Pyruvate dehydrogenase E1 component subunit alpha, testis-specific form, mitochondrial | 0.3 | 2 |
| Q8BMK4 | Cytoskeleton-associated protein 4 | 0.3 | 2 |
| Q6P9L3 | Bptf protein | 0.3 | 2 |
| Q7M6Y6 | Maestro heat-like repeat-containing protein family member 2B | 0.2 | 2 |
| Q8R3F0 | Larp2 protein | 0.2 | 2 |
| O70279 | Protein DGCR14 | 0.2 | 2 |

*^a^*Protein accession numbers from UniProtKB. *^b^*Protein name. *^c^*Ratio of the relative protein abundance between (A) control AJ and (B) 10 ppmF-treated A/J mice. Significant differences in protein abundance were considered when ratio ≤ 0.5 or ≥ 1.5. Ratio ≤ 0.5 means increase in group B in relation to group A and ratio ≥ 1.5 means decrease in group B in relation to group A. *^d^*Number of peptides identified.
