# Supplementary material for: Bone Response to Fluoride Exposure Is Influenced by Genetics
Source: PLoS One. 2014 Dec 11;9(12):e114343. doi: 10.1371/journal.pone.0114343 (PMC4263599; doi:10.1371/journal.pone.0114343)
Supplement: S5 Table — Complete list of identified proteins with differences in abundance in the comparison between control A/J and 50 ppmF-treated A/J mice. (DOCX) [file pone.0114343.s010.docx]

**Supplemental Table 5.** Identified proteins with differences in abundance in the comparison between control A/J and 50 ppmF-treated A/J mice.

| Acession Number*^a^* | **Protein*^b^*** | **Ratio*^c^*** | **Nº of peptides*^d^*** |
| --- | --- | --- | --- |
| E9PZP8 | Protein Herc1 hect domain and RCC1_like domain 1 | 1.9 | 2 |
| P08003 | Protein disulfide-isomerase A4 | 1.8 | 2 |
| P37040 | NADPH--cytochrome P450 reductase | 1.7 | 2 |
| P70696 | Histone H2B type 1-A | 1.7 | 2 |
| Q80Z68 | Protein Arhgap4 | 1.5 | 2 |
| Q69ZX6 | MORC family CW-type zinc finger protein 2A | 1.5 | 2 |
| Q5DU28 | Pecanex-like protein 2 | 1.5 | 2 |
| Q8BM55 | Transmembrane protein 214 | 1.5 | 2 |
| Q3V0G7 | GTPase-activating Rap/Ran-GAP domain-like protein 3 | 1.5 | 2 |
| Q9DBR2 | Protein FAM13C | 1.5 | 2 |
| Q6PDK8 | E3 ubiquitin-protein ligase DTX4 | 1.5 | 2 |
| Q7TQC8 | Eukaryotic translation initiation factor 2 alpha kinase 3 | 1.5 | 2 |
| Q9ERK4 | Exportin-2 | 1.5 | 2 |
| [Q5SXY1](http://www.uniprot.org/uniprot/Q5SXY1) | cytospin_B | 1.5 | 2 |
| F8WJD4 | symplekin | 1.5 | 2 |
| Q8K3P5 | CCR4-NOT transcription complex subunit 6 | 1.5 | 2 |
| Q9ES52 | Phosphatidylinositol 3,4,5-trisphosphate 5-phosphatase 1 | 0.4 | 2 |

*^a^*Protein accession numbers from UniProtKB. *^b^*Protein name. *^c^*Ratio of the relative protein abundance between (A) control AJ and (B) 50 ppmF-treated A/J mice. Significant differences in protein abundance were considered when ratio ≤ 0.5 or ≥ 1.5. Ratio ≤ 0.5 means increase in group B in relation to group A and ratio ≥ 1.5 means decrease in group B in relation to group A. *^d^*Number of peptides identified.
