# Supplementary material for: Bone Response to Fluoride Exposure Is Influenced by Genetics
Source: PLoS One. 2014 Dec 11;9(12):e114343. doi: 10.1371/journal.pone.0114343 (PMC4263599; doi:10.1371/journal.pone.0114343)
Supplement: S6 Table — Complete list of identified proteins with differences in abundance in the comparison between 10 ppmF-treated A/J and 50 ppmF-treated A/J mice. (DOCX) [file pone.0114343.s011.docx]

**Supplemental Table 6.** Identified proteins with differences in abundance in the comparison between 10 ppmF-treated A/J and 50 ppmF-treated A/J mice.

| **Acession Number*^a^*** | **Protein*^b^*** | **Ratio*^c^*** | **Nº of peptides*^d^*** |
| --- | --- | --- | --- |
| Q9D3E6 | Cohesin subunit SA-1 | 3.5 | 2 |
| Q78F42 | zinc finger protein 324 | 3.4 | 2 |
| Q80Z37 | E3 ubiquitin-protein ligase Topors | 3.4 | 2 |
| Q6P549 | Phosphatidylinositol 3,4,5-trisphosphate 5-phosphatase 2 | 3.3 | 2 |
| Q9JHI8 | NADPH oxidase 4 | 3.3 | 2 |
| Q9WTU0 | Lysine-specific demethylase PHF2 | 2.8 | 2 |
| O08550 | Histone-lysine N-methyltransferase 2B | 2.8 | 3 |
| Q6P5D3 | Putative ATP-dependent RNA helicase DHX57 | 2.5 | 2 |
| P55850 | Desmocollin-3 | 2.2 | 2 |
| E9Q0Y4 | Signal-induced proliferation-associated protein 1 | 2.1 | 2 |
| Q587J6 | LINE-1 type transposase domain-containing protein 1 | 1.6 | 2 |
| Q8CC12 | Codanin-1 | 1.5 | 2 |
| Q0GNC1 | Inverted formin-2 | 1.5 | 2 |
| B2RXM5 | Protein Sec14l5 | 1.5 | 2 |
| Q8BUP8 | Protein FAM43A | 1.5 | 2 |

*^a^*Protein accession numbers from UniProtKB. *^b^*Protein name. *^c^*Ratio of the relative protein abundance between (A) 10 ppmF-treated AJ and (B) 50 ppmF-treated A/J mice. Significant differences in protein abundance were considered when ratio ≤ 0.5 or ≥ 1.5. Ratio ≤ 0.5 means increase in group B in relation to group A and ratio ≥ 1.5 means decrease in group B in relation to group A. *^d^*Number of peptides identified.
