# Supplementary material for: Bone Response to Fluoride Exposure Is Influenced by Genetics
Source: PLoS One. 2014 Dec 11;9(12):e114343. doi: 10.1371/journal.pone.0114343 (PMC4263599; doi:10.1371/journal.pone.0114343)
Supplement: S7 Table — Complete list of identified proteins with differences in abundance in the comparison between control 129P3/J and control A/J mice. (DOCX) [file pone.0114343.s012.docx]

**Supplemental Table 7.** Identified proteins with differences in abundance in the comparison between control 129P3/J and control A/J mice.

| **Acession Number*^a^*** | **Protein*^b^*** | **Ratio*^c^*** | **Nº of peptides*^d^*** |
| --- | --- | --- | --- |
| Q9D3E6 | Cohesin subunit SA-1 | 3.8 | 2 |
| Q9Z123 | Semaphorin-4F | 3.8 | 2 |
| Q6PDK8 | E3 ubiquitin-protein ligase DTX4 | 3.7 | 2 |
| Q8R3N1 | Nucleolar protein 14 | 2.8 | 2 |
| Q9JHI8 | NADPH oxidase 4 | 2.7 | 2 |
| Q9WTU0 | Lysine-specific demethylase PHF2 | 2.6 | 3 |
| Q99PI5 | phosphatidate phosphatase LPIN2 | 2.4 | 2 |
| A2AMT1 | Filensin | 2.3 | 2 |
| Q9Z1N6 | Secreted frizzled-related sequence protein 4 | 2.3 | 2 |
| Q9JL61 | DNA-binding protein Rfx5 | 2.3 | 2 |
| Q7TNF8 | Peripheral-type benzodiazepine receptor-associated protein 1 | 2.3 | 2 |
| E9Q5G2 | Protein Pcdhb9 | 2.1 | 2 |
| O35348 | Acetylcholinesterase collagenic tail peptide | 2.1 | 2 |
| A2AQ25 | Sickle tail protein | 2.1 | 2 |
| Q8VHK9 | Probable ATP-dependent RNA helicase DHX36 | 2.1 | 2 |
| A3KMN0 | Zbtb16 protein | 2.1 | 2 |
| Q6PDG0 | Nup205 protein | 2.1 | 2 |
| A2AQ25 | Sickle tail protein | 2.0 | 3 |
| Q7TN74 | Neurabin-1 | 2.0 | 2 |
| Q9Z0X4 | cGMP-inhibited 3´,5´-cyclic phosphodiesterase A | 2.0 | 2 |
| Q9WU40 | Inner nuclear membrane protein Man1 | 2.0 | 2 |
| Q6XUX1 | Dual serine/threonine and tyrosine protein kinase | 1.9 | 2 |
| Q8K341 | Alpha-tubulin N-acetyltransferase | 1.9 | 2 |
| Q8C0T5 | Signal-induced proliferation-associated 1-like protein 1 | 1.9 | 2 |
| Q8VCV1 | Alpha/beta hydrolase domain-containing protein 17C | 1.9 | 2 |
| Q9GNC1 | Inverted formin-2 | 1.9 | 2 |
| Q99JF8 | PC4 and SFRS1-interacting protein | 1.9 | 2 |
| Q80Z37 | E3 ubiquitin-protein ligase Topors | 1.9 | 3 |
| P25119 | Tumor necrosis factor receptor superfamily member 1B | 1.9 | 2 |
| A2A5K6 | Zinc finger protein 335 | 1.8 | 2 |
| Q4KWH5 | 1-phosphatidylinositol 4´,5´-bisphosphate phosphodiesterase eta-1 | 1.8 | 2 |
| Q10470 | Beta-1,4-mannosyl-glycoprotein 4-beta-N-Acetylglucosaminyltransferase | 1.8 | 2 |
| P04370 | Myelin basic protein | 1.8 | 2 |
| Q3TAP4 | AP-5 complex subunit beta-1 | 1.8 | 2 |
| Q8BWJ3 | Phosphorylase b kinase regulatory subunit alpha, liver isoform | 1.8 | 2 |
| Q8BM55 | Transmembrane protein 214 | 1.8 | 2 |
| Q9JMH9 | Unconventional myosin-XVIIIa | 1.8 | 2 |
| Q6QIY3 | Sodium channel protein type 10 subunit alpha | 1.8 | 2 |
| Q8CIS7 | Polyductin | 1.8 | 2 |
| Q61423 | Potassium voltage-gated channel subfamily A member 4 | 1.7 | 2 |
| Q8C8K6 | Ataxin 7-like protein 2 | 1.7 | 2 |
| Q14B46 | Rhotekin-2 | 1.7 | 2 |
| Q91XQ0 | Dynein heavy chain 8, axonemal | 1.7 | 2 |
| Q5SWD9 | Pre-rRNA-processing protein TSR1 homolog | 1.7 | 2 |
| Q9QWV9 | Cyclin-T1 | 1.7 | 2 |
| Q3UWA6 | Heat-stable enterotoxin receptor | 1.7 | 2 |
| Q99LE6 | ATP-binding cassette sub-family F member 2 | 1.7 | 2 |
| P02088 | Hemoglobin subunit beta-1 | 1.7 | 5 |
| P02089 | Hemoglobin subunit beta-2 | 1.7 | 6 |
| Q8BL65 | Actin-binding LIM protein 2 | 1.7 | 2 |
| Q61879 | Myosin-10 | 1.7 | 3 |
| A2A870 | Fas-binding factor 1 | 1.7 | 2 |
| B2RRE4 | Zinc finger protein 518B | 1.7 | 2 |
| Q9QXL1 | Kinesin-like protein KIF21B | 1.7 | 2 |
| Q6VH22 | Intraflagellar transport protein 172 homolog | 1.7 | 3 |
| Q8CHP0 | Zinc finger CCCH domain-containing protein 3 | 1.6 | 2 |
| Q8C716 | Hemicentin 1 | 1.6 | 2 |
| Q059Y8 | DC-STAMP domain-containing protein 1 | 1.6 | 3 |
| Q6ZPI0 | Protein Jade-1 | 1.6 | 2 |
| P01942 | Hemoglobin subunit alpha | 1.6 | 6 |
| Q6PDM2 | Serine/arginine-rich splicing factor 1 | 1.6 | 2 |
| P97347 | Repetin | 1.6 | 2 |
| Q3TY86 | Apoptosis-inducing factor 3 | 1.6 | 2 |
| Q3TMV7 | Pyridine nucleotide-disulfide oxidoreductase domain-containing protein 1 | 1.6 | 2 |
| P60755 | MAM domain-containing glycosylphosphatidylinositol anchor protein 2 | 1.6 | 2 |
| E9QAP1 | Protein Zfp764 | 1.6 | 2 |
| Q9ERK4 | Exportin-2 | 1.6 | 2 |
| Q99PJ1 | protocadherin beta 15 | 1.6 | 2 |
| A3KGS3 | Ral GTPase-activating protein subunit alpha-2 | 1.6 | 2 |
| Q8K348 | Activin receptor type-1C | 1.6 | 2 |
| O55026 | Ectonucleoside triphosphate diphosphohydrolase 2 | 1.6 | 2 |
| O08638 | Myosin-11 | 1.6 | 2 |
| Q8CC52 | Eph receptor A7, isoform CRA_a | 1.6 | 2 |
| P59900 | EMILIN_3 | 1.6 | 2 |
| Q6URW6 | Myosin-14 | 1.6 | 2 |
| Q99K41 | EMILIN-1 | 1.6 | 2 |
| Q8K400 | Syntaxin-binding protein 5 | 1.6 | 2 |
| A2AJK6 | Chromodomain-helicase-DNA-binding protein 7 | 1.6 | 2 |
| Q8BIQ6 | Protein Zfp947 | 1.6 | 2 |
| K7N6U5 | Protein Vmn2r78 | 1.6 | 2 |
| E9PY16 | Protein Adap1 | 1.6 | 2 |
| A2AQH4 | BCL-6 corepressor-like protein 1 | 1.6 | 2 |
| O88866 | Hormonally up-regulated neu tumor-associated kinase | 1.6 | 2 |
| Q8K3I4 | Rab effector MyRIP | 1.6 | 2 |
| Q8BUH8 | Sentrin-specific protease 7 | 1.6 | 2 |
| Q60665 | Ski-like protein | 1.6 | 2 |
| Q9QZ04 | MAGE-like protein 2 | 1.6 | 2 |
| P30415 | NK-tumor recognition protein | 1.6 | 2 |
| P97855 | Ras GTPase-activating protein-binding protein 1 | 1.6 | 2 |
| D3Z183 | Major facilitator superfamily domain-containing protein 6 | 1.6 | 2 |
| Q8VHK1 | Caskin-2 | 1.6 | 2 |
| E9PVX6 | Protein Mki67 | 1.6 | 3 |
| Q9D7V2 | LysM and putative peptidoglycan-binding domain-containing protein 2 | 1.6 | 2 |
| Q80YR7 | Claspin | 1.6 | 2 |
| Q66JY0 | Wee1-like protein kinase 2 | 1.6 | 2 |
| Q5DTT3 | Protein FAM208B | 1.5 | 2 |
| Q7TQC3 | A11 integrin | 1.5 | 2 |
| Q8BMI0 | F-box only protein 38 | 1.5 | 3 |
| Q8K0D5 | Elongation factor G, mitochondrial | 1.5 | 2 |
| B2RXM5 | Protein SEC1415 | 1.5 | 2 |
| P40124 | Adenylyl cyclase-associated protein 1 | 1.5 | 2 |
| Q62381 | Tolloid-like protein 1 | 1.5 | 2 |
| P49645 | Transcription factor SOX-6 | 1.5 | 2 |
| O88811 | Signal transducing adapter molecule 2 | 1.5 | 2 |
| A2AJ88 | Patatin-like phospholipase domain-containing protein 7 | 1.5 | 2 |
| Q05512 | Serine/threonine-protein kinase MARK2 | 1.5 | 2 |
| Q91WL8 | WW domain-containing oxidoreductase | 1.5 | 2 |
| P63101 | 14-3-3 protein zeta/delta | 1.5 | 3 |
| J3QMK1 | Protein Gm4975 | 1.5 | 2 |
| Q922S8 | Kinesin-like protein KIF2C | 1.5 | 2 |
| Q9WV02 | RNA- binding motif protein, X chromosome | 1.5 | 3 |
| Q91VM5 | RNA- binding motif protein, X-linked-like-1 | 1.5 | 3 |
| A2AKX3 | Probable helicase senataxin | 1.5 | 2 |
| Q9D5Y1 | Coiled-coil domain-containing protein 39 | 1.5 | 2 |
| Q6ZWQ0 | Nesprin-2 | 1.5 | 2 |
| Q4VA53 | Sister chromatid cohesion protein PDS5 homolog B | 1.5 | 2 |
| Q0VBL1 | Tigger transposable element-derived protein 2 | 1.5 | 3 |
| Q8VDP2 | UPF0428 protein CXorf56 homolog | 1.5 | 2 |
| E9Q043 | Protein Fndc1 | 1.5 | 4 |
| O89026 | Roundabout homolog 1 | 1.5 | 2 |
| B1AR51 | Dynein heavy chain 9, axonemal | 1.5 | 2 |
| O35914 | Zinc finger protein basonuclin-1 | 1.5 | 2 |
| Q08274 | Dystrophia myotonica WD repeat-containing protein | 1.5 | 2 |
| E9Q774 | Protein Akap11 | 1.5 | 2 |
| Q6NZC7 | SEC23-interacting protein | 1.5 | 2 |
| Q6ZPZ3 | Zinc finger CCCH domain-containing protein 4 | 1.5 | 3 |
| P62204 | Calmodulin | 1.5 | 2 |
| Q5SSW2 | Proteasome activator complex subunit 4 | 1.5 | 2 |
| Q5HZI1 | Microtubule-associated tumor suppressor 1 homolog | 1.5 | 2 |
| Q61711 | bone sialoprotein 2 | 1.5 | 2 |
| Q9QXL1 | Kinesin-like protein KIF21B | 1.5 | 2 |
| Q8BJS8 | Mdm2-binding protein | 1.5 | 2 |
| P43276 | Histone H1.5 | 1.5 | 2 |
| P97798 | Neogenin | 1.5 | 2 |
| Q8BTM8 | Filamin-A | 1.5 | 2 |
| Q8BMJ2 | Leucyl-tRNA synthetase, cytoplasmic | 1.5 | 3 |
| Q9D7D7 | Claudin-23 | 1.5 | 2 |
| Q3URQ7 | Methenyltetrahydrofolate synthase domain-containing protein | 1.5 | 2 |
| Q8R4P4 | Transmembrane channel-like protein 2 | 1.5 | 3 |
| P46660 | Alpha-internexin | 1.5 | 2 |
| Q8CI51 | PDZ and LIM domain protein 5 | 1.5 | 2 |
| Q9JJL9 | Leukotriene B4 receptor 2 | 1.5 | 2 |
| P58871 | 182 kDa tankyrase-1-binding protein | 1.5 | 2 |
| Q810A7 | ATP-dependent RNA helicase DDX42 | 1.5 | 2 |
| D3YYQ8 | Dynein heavy chain 10, axonemal | 1.5 | 2 |
| D3YZG8 | Probable bifunctional methylenetetrahydrofolate dehydrogenase/cyclohydrolase 2 | 1.5 | 2 |
| Q497Q6 | Protein FAM228B | 1.5 | 2 |
| Q9QXK3 | Coatomer subunit gamma-2 | 1.5 | 2 |
| Q01149 | Collagen alpha-2(I) chain | 1.5 | 3 |
| Q99K95 | Protein RTF2 homolog | 1.5 | 2 |
| P98063 | Bone morphogenetic protein 1 | 1.5 | 3 |
| P20152 | Vimentin | 1.5 | 8 |
| Q99PP2 | Protein Zfp318 | 1.5 | 2 |
| Q91Y04 | protocadherin beta 15 | 1.5 | 2 |
| Q9QUS6 | Probable E3 ubiquitin-protein ligase MID2 | 1.5 | 2 |
| Q811S7 | Upstream-binding protein 1 | 1.5 | 2 |
| P59281 | Rho GTPase-activating protein 39 | 1.5 | 2 |
| Q69ZL1 | FYVE, RhoGEF and PH domain-containing protein 6 | 1.5 | 3 |
| Q9D2L9 | Protein FAM111A | 1.5 | 2 |
| P27786 | Steroid 17-alpha-hydroxylase/17,20 lyase | 1.5 | 2 |
| Q3V3R4 | Integrin alpha-1 | 1.5 | 2 |
| Q9ES03 | T-box transcription factor TBX20 | 1.5 | 3 |
| P11087 | Collagen alpha-1(I) chain | 1.5 | 6 |

*^a^*Protein accession numbers from UniProtKB. *^b^*Protein name. *^c^*Ratio of the relative protein abundance between (A) control 129P3/J and (B) control A/J mice. Significant differences in protein abundance were considered when ratio ≤ 0.5. Ratio of ≤ 0.5 means increase in group B in relation to group A. *^d^*Number of peptides identified.
