# Supplementary material for: Bone Response to Fluoride Exposure Is Influenced by Genetics
Source: PLoS One. 2014 Dec 11;9(12):e114343. doi: 10.1371/journal.pone.0114343 (PMC4263599; doi:10.1371/journal.pone.0114343)
Supplement: S8 Table — Complete list of identified proteins with differences in abundance in the comparison between 10 ppmF-treated 129P3/J and 10 ppmF-treated A/J mice. (DOCX) [file pone.0114343.s013.docx]

**Supplemental Table 8.** Identified proteins with differences in abundance in the comparison between 10 ppmF-treated 129P3/J and 10 ppmF-treated A/J mice.

| **Acession Number*^a^*** | **Protein*^b^*** | **Ratio*^c^*** | **Nº of peptides*^d^*** |
| --- | --- | --- | --- |
| Q6P549 | Phosphatidylinositol 3,4,5-trisphosphate 5-phosphatase 2 | 2.0 | 2 |
| O88509 | DNA (cytosine-5)-methyltransferase 3B | 1.7 | 2 |
| E9Q5L8 | Protein Glyatl3 | 1.7 | 2 |
| Q80Z37 | E3 ubiquitin-protein ligase Topors | 1.6 | 2 |
| O08550 | Histone-lysine N-methyltransferase 2B | 1.6 | 2 |
| Q0GNC1 | Inverted formin-2 | 1.5 | 2 |
| Q9JHI8 | NADPH oxidase 4 | 1.5 | 2 |
| Q8C419 | Probable G-protein coupled receptor 158 | 1.5 | 2 |
| O88491 | Histone-lysine N-methyltransferase, H3 lysine-36 and H4 lysine-20 specific | 0.5 | 2 |
| Q9R0L6 | Pericentriolar material 1 protein | 0.5 | 2 |
| Q6PDF3 | Putative transporter SVOPL | 0.4 | 2 |
| P62204 | Calmodulin | 0.4 | 2 |
| A6H6E9 | Tetratricopeptide repeat protein 23-like | 0.4 | 2 |
| Q6NWW5 | Kinesin-like protein KIF24 | 0.4 | 2 |

*^a^*Protein accession numbers from UniProtKB. *^b^*Protein name. *^c^*Ratio of the relative protein abundance between (A) 10 ppmF-treated 129P3/J and (B) 10 ppmF-treated A/J mice. Significant differences in protein abundance were considered when ratio ≤ 0.5 or ≥ 1.5. Ratio ≤ 0.5 means increase in group B in relation to group A and ratio ≥ 1.5 means decrease in group B in relation to group A. *^d^*Number of peptides identified.
