# Supplementary material for: Bone Response to Fluoride Exposure Is Influenced by Genetics
Source: PLoS One. 2014 Dec 11;9(12):e114343. doi: 10.1371/journal.pone.0114343 (PMC4263599; doi:10.1371/journal.pone.0114343)
Supplement: S9 Table — Complete list of identified proteins with differences in abundance in the comparison between 50 ppmF-treated 129P3/J and 50 ppmF-treated A/J mice. (DOCX) [file pone.0114343.s014.docx]

**Supplemental Table 9.** Identified proteins identified with differences in abundance in the comparison between 50 ppmF-treated 129P3/J and 50 ppmF-treated A/J mice.

| **Acession Number*^a^*** | **Protein*^b^*** | **Ratio*^c^*** | **Nº of peptides*^d^*** |
| --- | --- | --- | --- |
| Q3TIU4 | 2´,5´-phosphodiesterase 12 | 1.8 | 2 |
| Q8R3P9 | Ankyrin repeat domain-containing protein 32 | 1.6 | 2 |
| Q8K4G5 | actin_binding LIM protein 2 isoform 1 | 1.6 | 2 |
| P62932 | F-box only protein 40 | 1.5 | 2 |
| Q8QZV7 | Protein asunder homolog | 0.4 | 2 |

*^a^*Protein accession numbers from UniProtKB. *^b^*Protein name. *^c^*Ratio of the relative protein abundance between (A) 50 ppmF-treated 129P3/J and (B) 50 ppmF-treated A/J mice. Significant differences in protein abundance were considered when ratio ≤ 0.5 or ≥ 1.5. Ratio ≤ 0.5 means increase in group B in relation to group A and ratio ≥ 1.5 means decrease in group B in relation to group A. *^d^*Number of peptides identified.
